# DUSP11-mediated control of 5′-triphosphate RNA regulates RIG-I sensitivity

**DOI:** 10.1101/gad.340604.120

**Published:** 2020-12-01

**Authors:** Joon H. Choi, James M. Burke, Kayla H. Szymanik, Upasana Nepal, Anna Battenhouse, Justin T. Lau, Aaron Stark, Victor Lam, Christopher S. Sullivan

**Affiliations:** Department of Molecular Biosciences, LaMontagne Center for Infectious Disease, The University of Texas at Austin, Austin Texas 78712, USA

**Keywords:** DUSP11, RIG-I signaling, inflammation, innate immunity, noncoding RNA, tumor–stromal interaction, virus infection

## Abstract

Deciphering the mechanisms that regulate the sensitivity of pathogen recognition receptors is imperative to understanding infection and inflammation. Here we demonstrate that the RNA triphosphatase dual-specificity phosphatase 11 (DUSP11) acts on both host and virus-derived 5′-triphosphate RNAs rendering them less active in inducing a RIG-I-mediated immune response. Reducing DUSP11 levels alters host triphosphate RNA packaged in extracellular vesicles and induces enhanced RIG-I activation in cells exposed to extracellular vesicles. Virus infection of cells lacking DUSP11 results in a higher proportion of triphosphorylated viral transcripts and attenuated virus replication, which is rescued by reducing RIG-I expression. Consistent with the activity of DUSP11 in the cellular RIG-I response, mice lacking DUSP11 display lower viral loads, greater sensitivity to triphosphorylated RNA, and a signature of enhanced interferon activity in select tissues. Our results reveal the importance of controlling 5′-triphosphate RNA levels to prevent aberrant RIG-I signaling and demonstrate DUSP11 as a key effector of this mechanism.

## Abstract (precis)

In this study, Choi et al. set out to elucidate the physiological role of RNA triphosphatase dual-specificity phosphatase 11 (DUSP11) in the innate immune response. Using in vivo and in vitro experiments, the authors describe the importance of controlling 5′-triphosphate RNA levels to prevent aberrant RIG-I signaling and demonstrate DUSP11 as a key effector of this mechanism.

Life is a great balancing act with overlapping and unique molecular controls required to maintain homeostasis (Kotas and Medzhitov 2015; tenOever 2016). Host detection of pathogens must be properly tuned to promote rapid and effective responses when an actual pathogen is detected, but must not be so sensitive as to trigger an inflammatory response when no pathogen is present (Medzhitov and Janeway 2002; Roers et al. 2016). RIG-I is a pattern recognition receptor (PRR) that triggers the antiviral cytokine type I interferon (IFN) response (Yoneyama et al. 2004; Kato et al. 2005; Gack et al. 2007). Major pathogen-associated molecular patterns (PAMPs) that activate RIG-I are structured or double-stranded RNAs that possess a 5′-triphosphate moiety, a hallmark of viral RNA-dependent RNA polymerase (RdRP) transcripts that are expressed during the course of infection (Hornung et al. 2006; Pichlmair et al. 2006). However, host transcripts, at least initially, also possess a 5′-triphosphate. In particular, many host transcripts generated by RNA polymerase III (RNAP III) lack a 5′ capping mechanism (Dieci et al. 2013). A series of recent studies indicate that some of these endogenous RNAP III transcripts can function as damage-associated molecular patterns (DAMPs), which are recognized by RIG-I (Boelens et al. 2014; Nabet et al. 2017; Chiang et al. 2018; Zhao et al. 2018). To avoid autoinflammatory pathology, the RIG-I response must therefore be carefully regulated. Indeed, several upstream and downstream protein modulators of RIG-I signaling have already been identified (Chiang et al. 2014). However, host mechanisms that directly control the 5′-triphosphate proinflammatory capacity of PAMP or DAMP transcripts are not well understood.

DUSP11 is an RNA triphosphatase that we and others have previously demonstrated to be active on the 5′ ends of structured host and viral transcripts (Deshpande et al. 1999; Burke et al. 2016; Burke and Sullivan 2017; Kincaid et al. 2018; Vabret et al. 2019). The 5′-triphosphates of hepatitis C virus (HCV) RNAs are converted to monophosphates rendering them susceptible to exonuclease XRN-mediated attack (Amador-Cañizares et al. 2018; Kincaid et al. 2018). A different outcome arises when triphosphorylated RNAP III viral precursor microRNAs (miRNAs) are processed by DUSP11. These viral miRNAs require DUSP11 for full silencing activity via stable loading of 5′-monophosphate miRNAs into the RNA-induced silencing complex (RISC) (Burke et al. 2016). Additionally, the 5′-triphosphate status of several host RNAP III noncoding RNA transcripts including vault RNAs (vtRNAs), Alu SINE RNAs, Y RNAs, and RMRP RNA are also modulated by DUSP11 (Burke et al. 2016; Zhao et al. 2018; Vabret et al. 2019). The ability of DUSP11 to convert the 5′ end of diverse structured and partially double-stranded host and viral transcripts into monophosphates opens up the possibility that DUSP11 can alter the immunostimulatory activity of endogenous and exogenous triphosphorylated transcripts (Burke and Sullivan 2017).

Although DUSP11 can convert structured triphosphate RNAs into monophosphates (Burke and Sullivan 2017), and DUSP11 has been implicated as being reduced during infection by some viruses (Zhao et al. 2018; Vabret et al. 2019), the physiological role of DUSP11 in the innate immune response is poorly understood. Here we test the hypothesis that DUSP11 is a modulator of the RNA-induced innate immune response. Our data demonstrate that tuning the 5′-triphosphate levels of both endogenous host and exogenous viral transcripts is a regulator of RIG-I signaling and that DUSP11 is a key mediator of 5′-triphosphate balance.

## Results

### DUSP11 modulates RIG-I signaling sensitivity to liposomal 5′-triphosphate RNAs

Among the host defense machineries that sense invading pathogens, RIG-I is the key PRR that recognizes the 5′-triphosphate or diphosphate moiety of double-stranded RNA transcripts and in turn activates the antiviral immune defense signaling pathway (Hornung et al. 2006). Previous work demonstrated that DUSP11 sequentially removes the γ and β phosphates of triphosphorylated structured host and viral transcripts leaving 5′-monophosphates (Deshpande et al. 1999; Burke et al. 2016; Kincaid et al. 2018). This led us to hypothesize that DUSP11 can modulate the immunogenicity of triphosphorylated transcripts (Burke and Sullivan 2017) and alter the sensitivity of these transcripts to detection by RIG-I. To begin to test this hypothesis, we first looked for evidence of enhanced interferon-mediated gene expression. Although our previous RNA-seq experiments (Burke et al. 2016) did not display a strong signal of interferon response in HEK293T or A549 cells lacking DUSP11, we re-examined this possibility using RT-qPCR on early passage normal human dermal fibroblasts (NHDF) with reduced DUSP11 and A549 cells absent of DUSP11. While the levels for interferon transcripts were at the limit of detection, we observed enhanced expression of *IL-6* mRNA in NHDF cells with reduced DUSP11, and in both NHDF and A549 lineages, cells deficient of DUSP11 express slightly increased transcript levels of interferon-stimulated gene (ISG) 15 (Supplemental Fig. S1A,B). Combined with our previous work (Burke et al. 2016), these data do not support a background of strong antiviral response in cultured cells with reduced DUSP11, but are consistent with a subtle enhanced predisposition of these cells to undergo RIG-I signaling.

**Figure 1.**
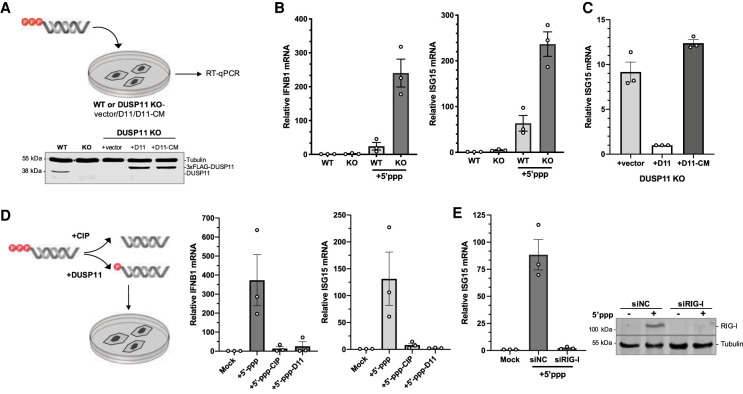
DUSP11 modulates RIG-I signaling sensitivity to liposomal 5′-triphosphate RNAs. (*A*) Schematic diagram of the 5′-ppp-RNA transfection assay, and immunoblot analysis of A549 parental wild-type (WT) and DUSP11 knockout (KO) cells transduced with pLenti empty vector (vector), DUSP11-3xFLAG (D11), or DUSP11-3xFLAG-catalytic mutant (D11-CM). A549 WT or DUSP11 knockout cells (12-well) were transfected with 5–10 ng of in vitro transcribed 5′-ppp-RNA for 18 h posttransfection. (*B*) RT-qPCR analysis of IFNB1 and ISG15 mRNA normalized to GAPDH mRNA in 5′-ppp-RNA transfected in WT and DUSP11 KO cells as in (*A*). Results are presented relative to mock-transfected WT cells. (*C*) RT-qPCR analysis of ISG15 mRNA normalized to GAPDH mRNA in DUSP11 KO cells stably expressing empty vector, DUSP11, or DUSP11 catalytic mutant as in *A*, transfected with 5′-ppp-RNA. Results are presented relative to those of DUSP11-expressing cells (+D11). (*D*) RT-qPCR analysis of IFNB1 and ISG15 mRNA normalized to GAPDH mRNA in DUSP11 knockout cells transfected with 5′-ppp-RNA pretreated with or without calf intestinal phosphatase (CIP) or in vitro-translated DUSP11-core. Results are presented relative to mock-treated DUSP11 knockout cells. (*E*) RT-qPCR analysis (*left*) of ISG15 mRNA normalized to GAPDH mRNA in DUSP11 knockout cells transfected with negative control siRNA (siNC) or siRNA targeting RIG-I (siRIG-I) and subsequently transfected with 5′-ppp-RNA, and immunoblot analysis (*right*) assessing siRIG-I knockdown efficiency of A549 cells transfected with or without 5′ppp-RNA. Data are derived from *n* = 3 independent replicates in *B*–*E* and are presented as mean ± SEM.

We next studied the effects of DUSP11 on exogenously introduced in vitro transcribed RNAs (Fig. 1A). We used the 5′ untranslated region (UTR) of the HCV RNA, as its expression is immunostimulatory (Saito et al. 2007). Additionally, we and others had previously demonstrated triphosphatase activity of DUSP11 on HCV RNAs during infection (Amador-Cañizares et al. 2018; Kincaid et al. 2018). Consistent with RIG-I induction, cationic-lipid-mediated transfection of HCV 5′-triphosphate RNA (referred to here as “5′-ppp-RNA”) into the A549 adenocarcinoma human lung epithelial cell line resulted in the induction of both IFNB1 cytokine and *ISG15* mRNA transcripts (Fig. 1B). Under conditions where DUSP11 protein levels are abolished due to CRISPR-mediated knockout, transfection of 5′-ppp-RNA results in enhanced induction of *IFNB1* and *ISG15* mRNA (Fig. 1B). Consistent with RIG-I being an interferon-stimulated gene (Matsumiya and Stafforini 2010), RIG-I protein levels were also further induced in cells with reduced DUSP11 (Supplemental Fig. S1C). To determine whether this regulation was dependent on the catalytic triphosphatase activity of DUSP11, we used A549 DUSP11 knockout cells stably reconstituted with a control empty vector (vector), wild-type DUSP11 (D11), or the catalytically inactive mutant DUSP11 (Cys152Ser, D11-CM) (Fig. 1A; Yuan et al. 1998; Deshpande et al. 1999; Burke et al. 2016). Upon transfection of 5′-ppp-RNA, expression of wild-type DUSP11, but not the empty vector or catalytic mutant DUSP11, reduced *ISG15* mRNA induction (Fig. 1C). Consistent with DUSP11 directly reducing the visibility of RNAs to RIG-I, pretreatment of 5′-ppp-RNA with calf intestinal phosphatase (CIP) or the purified catalytic core of DUSP11 repeatably resulted in reduced induction of *IFNB1* and *ISG15* transcripts (Fig. 1D). To test the requirement of RIG-I for ISG induction, we used siRNA-mediated knockdown of RIG-I. Cells with reduced RIG-I displayed significantly reduced induction of *ISG15* transcripts (Fig. 1E). Together, these results indicate that DUSP11 modulates RIG-I signaling sensitivity via its catalytic phosphatase activity on 5′-triphosphate PAMP RNA.

### DUSP11 alleviates the interferon response mediated by tumor–fibroblast cell interaction

**Figure 2.**
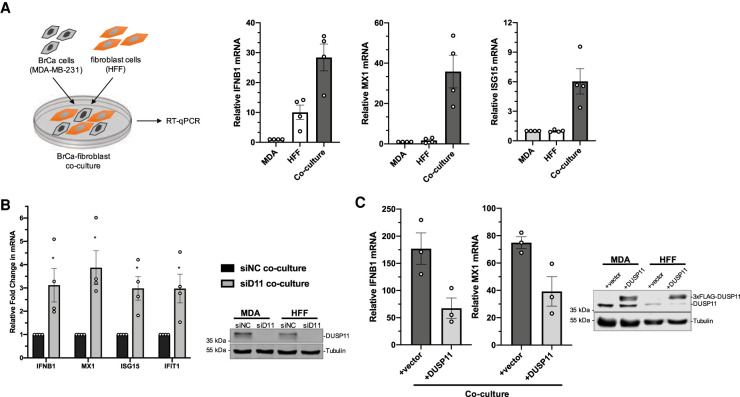
DUSP11 alleviates the interferon response mediated by tumor–fibroblast cell interaction. (*A*) Schematic diagram of coculture assay (*left*), and RT-qPCR analysis (*right*) of IFNB1, MX1, and ISG15 mRNA induction normalized to GAPDH mRNA in MDA-MB-231 breast cancer (BrCa) cells and HFF fibroblast cells either in monoculture or coculture. Cells were cultured for 60–72 h and RNA lysates were collected for RT-qPCR analysis. RT-qPCR results are presented relative to monocultured MDA-MB-231 cells. (*B*) RT-qPCR analysis (*left*) of IFNB1 and ISG mRNA transcripts (MX1, ISG15, and IFIT1 mRNA) comparing fold change of cocultured cells silenced of DUSP11 (siD11 coculture) relative to control coculture (siNC coculture), and immunoblot analysis (*right*) assessing siRNA knockdown of DUSP11 in MDA-MB-231 and HFF cells treated with negative control siRNA (siNC) or siRNA targeting DUSP11 (siD11). (*C*) RT-qPCR analysis (*left*) of IFNB1 and MX1 mRNA normalized to GAPDH mRNA in cocultured MDA-MB-231 and HFF cells transduced with pLenti empty vector (vector) or pLenti-DUSP11-3xFLAG (D11), and immunoblot analysis (*right*) of endogenous and 3xFLAG-tagged DUSP11 in whole-cell lysates. RT-qPCR results are presented relative to those of monocultured MDA-MB-231 vector cells. Data are derived from *n* = 4 independent replicates in *A* and *B* and *n* = 3 independent replicates in *C*. In all panels, data are presented as mean ± SEM. (*) *P* < 0.05 (two-tailed Student's *t*-test).

We next wanted to determine whether DUSP11 alters cellular susceptibility to endogenous RIG-I DAMP RNAs. Accumulating evidence indicates that during cellular stress conditions, endogenous RNA transcripts can be sensed by host PRRs and trigger inflammation (Tarallo et al. 2012; Boelens et al. 2014; White et al. 2014; Chiappinelli et al. 2015; Liddicoat et al. 2015; Roulois et al. 2015; Nabet et al. 2017; Chiang et al. 2018; Chung et al. 2018; Dhir et al. 2018; Zhao et al. 2018; Rehwinkel and Gack 2020). In the tumor microenvironment, extracellular vesicles (EVs) from stromal fibroblasts can deliver RIG-I-stimulatory noncoding RNAs to adjacent tumor cells and trigger the interferon response (Boelens et al. 2014; Nabet et al. 2017). The resulting proinflammatory signaling pathway in the recipient tumor cells promotes more aggressive tumors (Boelens et al. 2014). To determine whether DUSP11 would influence RIG-I sensitivity toward endogenous DAMP RNAs, we cocultured human foreskin fibroblasts (HFF) with breast cancer cells (MDA-MB-231), with or without knockdown of DUSP11, and assayed for enhanced interferon transcript induction. Similar to previous results (Boelens et al. 2014; Nabet et al. 2017), coculturing of HFFs with MDA-MB-231 cells resulted in the activation of the interferon response as evidenced by enhanced expression of *IFNB1*, *MX1*, and *ISG15* transcripts (Fig. 2A). While silencing *DUSP11* expression in individual monocultures of HFF and MDA-MB-231 cells showed no significant differences in *IFNB1* transcript levels, which were near the limit of detection (Supplemental Fig. S2A), we observed enhanced induction of *IFNB1*, *MX1*, *ISG15*, and *IFIT1* transcripts upon coculture of cells with reduced DUSP11 (Fig. 2B). Conversely, overexpression of DUSP11 reduced the magnitude of the interferon response (Fig. 2C). Although *IFNB1* transcript levels were slightly induced when DUSP11 was knocked down in just the HFF cells during coculture, *IFNB1* enhancement was greatest when both MDA-MB-231 and HFF cells were silenced for *DUSP11* expression (Supplemental Fig. S2B). These data demonstrate that DUSP11 can dampen the interferon response that is activated by the interaction between tumor and fibroblast cells.

**Figure 3.**
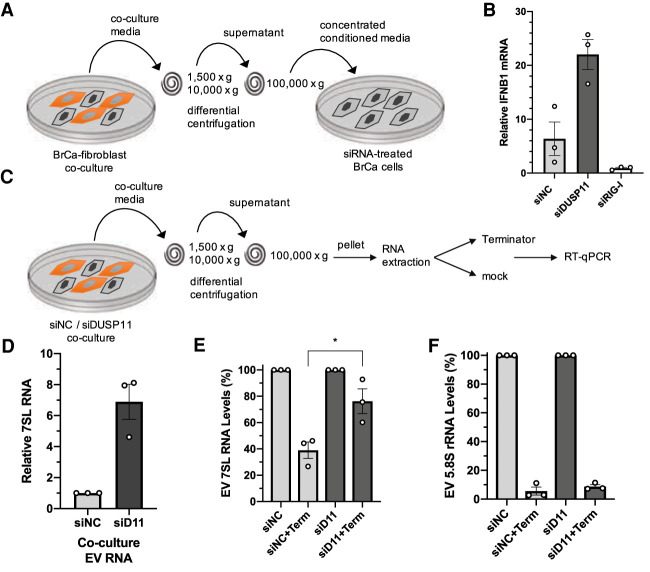
Reducing DUSP11 increases 7SL RNA 5′-triphosphate levels in tumor-fibroblast extracellular vesicles. (*A*) Schematic diagram of the coculture conditioned media transfer assay. EV-containing conditioned media was concentrated from coculture media by differential centrifugation and transferred to MDA-MB-231 breast cancer (BrCa) cells silenced for DUSP11 or RIG-I. (*B*) RT-qPCR analysis of IFNB1 mRNA in siRNA-treated MDA-MB-231 cells incubated in concentrated coculture conditioned media as in *A*. Results are presented relative to those of untreated mock MDA-MB-231 cells. (*C*) Schematic diagram of the 5′ end characterization RT-qPCR assay of 7SL RNA isolated from coculture EVs. EV-containing pellets were collected from coculture conditioned media by differential centrifugation. Extracted RNA was treated with DNase I followed by treatment with or without 5′-monophosphate-specific exonuclease Terminator. (*D*) RT-qPCR analysis of 7SL RNA in EV RNA extracted from siNC or siDUSP11 (siD11) cocultured conditioned media. Results are presented relative to siNC coculture with 5S rRNA as the endogenous control. (*E*) RT-qPCR analysis of 7SL RNA in EV RNA isolated from siNC or siDUSP11 (siD11) cocultured conditioned media, treated with or without Terminator. Results are presented relative to mock-treated siNC coculture with 5S rRNA as endogenous control. (*F*) RT-qPCR analysis of 5.8S rRNA in EV RNA isolated from siNC or siDUSP11 (siD11) cocultured conditioned media, treated with or without Terminator. Results are presented relative to mock-treated siNC coculture with 5S rRNA as endogenous control. Data are derived from *n* = 3 independent replicates in *B* and *D*–*F*. In all panels, data are presented as mean ± SEM. (*) *P* < 0.05 (two-tailed Student's *t*-test).

Given that our above data (Fig. 1) show that DUSP11 can reduce the sensitivity of RIG-I signaling triggered by transfected PAMP RNA, we asked if DUSP11 could also alter RIG-I signaling to cellular-generated DAMP RNA. Extracellular vesicles from cocultured fibroblast and tumor cells activate RIG-I in tumor cells via delivery of unshielded triphosphorylated 7SL RNA (Boelens et al. 2014; Nabet et al. 2017). Therefore, we applied EV-enriched conditioned media from cocultured cells onto MDA-MB-231 cancer cells and assayed for induction of *IFNB1* transcripts (Fig. 3A; Supplemental Fig. S2C). These data show that, as expected, treatment with EV-enriched conditioned media induces *IFNB1* mRNA expression (Fig. 3B). Knocking down *DUSP11* levels in the recipient cancer cells significantly enhanced the magnitude of *IFNB1* transcript induction (Fig. 3B; Supplemental Fig. S2D). As a control, knockdown of RIG-I showed the opposite effect resulting in reduced induction of IFN transcripts, demonstrating that cocultured conditioned media requires RIG-I to induce IFN transcripts (Fig. 3B; Supplemental Fig. S2D). These findings demonstrate that DUSP11 modulates RIG-I sensitivity in cells receiving incoming cell-generated DAMP RNAs.

We next asked whether DUSP11 could alleviate the immunogenicity of DAMP RNAs packaged into and secreted via extracellular vesicles. To determine whether reduced DUSP11 expression results in altered 7SL RNA levels in extracellular vesicles, we used siRNA to silence *DUSP11* and then cocultured HFF and MDA-MB-231 cells, harvested EVs, and assayed for 7SL RNA levels via RT-qPCR. These data showed an approximately sevenfold increase in the amount of 7SL RNA (relative to 5S rRNA) in EV preparations from cocultured cells with reduced DUSP11 levels (Fig. 3D). In contrast, 7SL RNA levels collected from whole cells showed a more modest increase in the DUSP11-silenced cocultures (1.8-fold) (Supplemental Fig. S2E), consistent with the effect of DUSP11 being greater on RNA cargo that is packaged in EVs.

Next, we examined whether DUSP11 alters the 5′ phosphate status of 7SL RNA in EVs. To do this, we harvested concentrated EV samples from the cocultured cells that had siRNA-mediated knockdown of DUSP11 expression. Purified RNAs from concentrated EV samples were then treated with 5′-monophosphate-specific Terminator exonuclease to indirectly estimate the fraction of 7SL RNAs that are triphosphorylated and resistant to Terminator (Fig. 3C). EV 7SL RNA from cells transfected with nontarget negative control siRNAs showed partial (∼60%) susceptibility to Terminator treatment, consistent with at least 60% of 7SL RNA having a 5′-monophosphate. Notably, Terminator treatment only reduced 7SL RNA levels from cells with siRNA-mediated knockdown of DUSP11 by ∼25%. This significant reduction in Terminator susceptibly is consistent with DUSP11 dephosphorylating a substantial portion of EV 7SL RNA. As previously reported (Burke et al. 2016), the endogenous control 5′-triphosphate 5S rRNA was unaffected by reduced DUSP11 levels (Supplemental Fig. S2F) while the positive control 5′-monophosphate 5.8S rRNA displayed susceptibility to Terminator treatment regardless of DUSP11 (Fig. 3E; Supplemental Fig. S2F). We conclude that DUSP11 can control the 5′-triphosphate status and abundance of DAMP RNAs packaged into EVs. Combined, these findings demonstrate that DUSP11 can affect DAMP RNA biology in both EV-producing and recipient cells.

### DUSP11 catalytic activity promotes RNA virus replication

**Figure 4.**
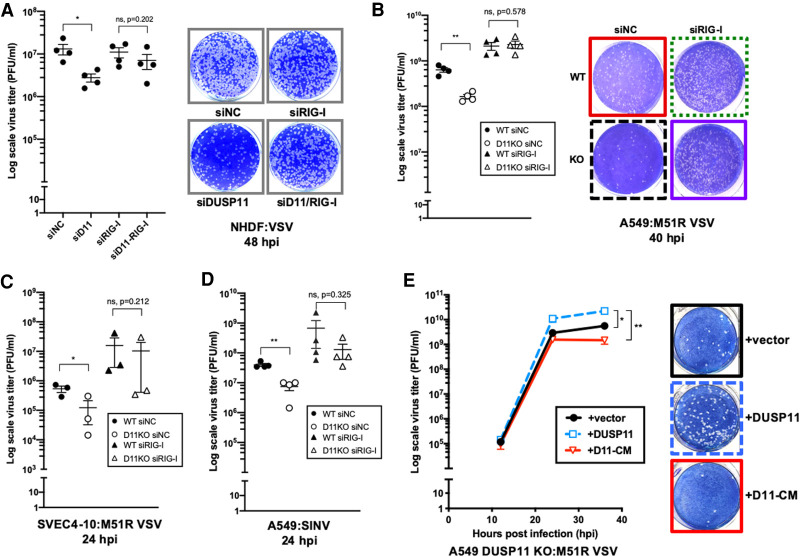
DUSP11 catalytic activity promotes RNA virus replication. (*A*) VSV viral titer of negative control siRNA (siNC), siRNA targeting DUSP11 (siD11), or RIG-I (siRIG-I)-treated NHDF cells determined by plaque assay (*left*), and representative image of plaque assay of cells at 48 h postinfection (hpi) (*right*). Cells treated with siRNA were infected with WT VSV at MOI of 0.25 PFU/cell and virus supernatant was collected 48 hpi for plaque assay analysis. (*B*) M51R VSV viral titer of siNC or siRIG-I-treated A549 WT and DUSP11 KO cells determined by plaque assay (*left*), and representative image of plaque assay of cells at 40 hpi (*right*). Cells treated with siRNA were infected with M51R VSV at MOI of 0.05 PFU/cell. (*C*) M51R VSV viral titer of siNC or siRIG-I-treated SVEC4-10 WT and DUSP11 KO cells determined by plaque assay. Cells were infected with M51R VSV at MOI of 0.1 PFU/cell for 24 h. (*D*) SINV viral titer of siNC or siRIG-I-treated A549 WT and DUSP11 KO cells determined by plaque assay. Cells treated with siRNA were infected with SINV at MOI of 0.05 PFU/cell for 24 h. (*E*) M51R VSV viral titer of A549 DUSP11 knockout cells reconstituted with empty vector (vector), DUSP11 (DUSP11) or catalytic mutant DUSP11 (D11-CM) as in Figure 1A determined by plaque assay (*left*), and representative image of plaque assay of cells at 36 hpi (*right*). Cells were infected with M51R VSV at MOI of 0.05 PFU/cell and virus supernatant was collected 12, 24, and 36 hpi for plaque assay analysis. Data are derived from *n* = 4 independent replicates in *A*, *B*, and *D*), and *n* = 3 independent replicates in *C* and *E*. In all panels, data are presented as mean ± SEM. (*) *P* < 0.05; (**) *P* < 0.01 (two-tailed Student's *t*-test).

Given that our above data demonstrate that DUSP11 dampens RIG-I-mediated antiviral signaling, we next investigated whether exogenous viral transcripts expressed during the course of infection are altered by the triphosphatase activity of DUSP11. We hypothesized that for some viruses, DUSP11 may be proviral by reducing the visibility of their transcripts to RIG-I. To investigate this, we examined RNA viruses known to express viral RNAs that are recognized by RIG-I (Kell and Gale 2015). First, we analyzed a negative-strand RNA virus, vesicular stomatitis virus (VSV) (Kato et al. 2005). When DUSP11 expression was reduced by siRNA-mediated knockdown, infection of early passage NHDF cells with VSV resulted in an approximately fivefold to 13-fold reduction in virus replication (Fig. 4A; Supplemental Figs. S3A, S4C).

To determine whether these results apply in other experimental systems with more facile knockout genetics, we examined infection in tumor cell lines and an immortalized cell line. A549 cells are known to have a partially intact interferon response (Spann et al. 2004; Nakatsu et al. 2006) and we had previously generated stable lines lacking DUSP11 expression (Burke et al. 2016). VSV infection of A549 DUSP11 knockout cells resulted in a nonsignificant trend of decrease in virus yield, albeit the magnitude of this effect was subtle (approximately twofold) (Supplemental Fig. S3B). To determine whether the DUSP11 knockout cells have an altered antiviral response using a more sensitive assay, we infected cells with the M51R mutant VSV. VSV M51R is defective for inhibiting host gene expression and, in turn, induces a stronger interferon response (Ahmed et al. 2003; Franz et al. 2018). Infection of A549 DUSP11 knockout cells (each pretreated with irrelevant negative control siRNA for a related experimental series described below) with M51R VSV resulted in a significant defect in infectious virus yield as compared with A549 wild-type cells (approximately fourfold) (Fig. 4B). Highly similar results were observed from a separate A549 DUSP11 knockout clone (Supplemental Fig. S3C). We obtained similar results (approximately fourfold decreased virus replication) when comparing infection of SV40-immortalized murine SVEC4-10 DUSP11 knockout cells to wild-type SVEC4-10 cells (each pretreated with irrelevant negative control siRNAs) (Fig. 4C; Supplemental Fig. S3D). However, overt differences in WT or M51R VSV replication were not observed in the HEK293 background, likely due to the highly attenuated antiviral response of HEK293 cells (Supplemental Fig. S3E; Reynolds 2006; Linehan et al. 2018; Thomsen et al. 2019). Combined, the above data demonstrate that reducing DUSP11 in cells of human or murine origin can promote partial resistance to virus replication.

VSV is a member of the Rhabdoviridae family with multiple members known to induce a RIG-I response (Faul et al. 2009). To determine whether DUSP11 affects replication of other virus families, we infected A549 wild-type or DUSP11 knockout cells with sindbis virus (SINV), a positive-strand togavirus that also expresses viral transcripts sensed by RIG-I (Akhrymuk et al. 2016), or herpes simplex 1 (HSV-1), a DNA virus. Similar to VSV, DUSP11 knockout cells (each pretreated with irrelevant negative control siRNAs) that were infected with SINV displayed a significant reduction (approximately fivefold) in infectious virus yield (Fig. 4D). Unlike for VSV M51R and SINV, we did not see an overt difference for HSV-1 when we infected WT or DUSP11 knockout A549 cells at the single time point and MOI that we examined (Supplemental Fig. S3F). We conclude that the reduced levels of DUSP11 negatively affect the replication of diverse RNA viruses known to be susceptible to RIG-I.

Although there are no known biochemical activities of DUSP11 other than phosphatase activity, it was formally possible that DUSP11 had unknown activities that could account for its role in promoting virus infection (Burke and Sullivan 2017). Therefore, we assayed A549 DUSP11 knockout cell lines with the restored wild-type DUSP11 (D11) or the catalytically inactive mutant of DUSP11 (D11-CM) for differences in susceptibility to M51R VSV infection. Infection of cells expressing wild-type DUSP11 resulted in an approximately fourfold enhanced virus replication over control empty vector-expressing cells and an ∼15-fold enhanced virus replication over cells expressing the DUSP11 catalytic mutant (Fig. 4E). These results demonstrate that expression of wild-type but not catalytic mutant DUSP11 is able to rescue the virus replication defect of DUSP11 knockout cells, consistent with DUSP11 phosphatase activity accounting for its proviral activity.

### DUSP11 promotes RNA virus replication by dephosphorylating viral RNA PAMPs

Our results demonstrating reduced virus replication in cells lacking DUSP11 are consistent with enhanced RIG-I signaling in these cells. To test the hypothesis that DUSP11-deficient cells have reduced viral infection due to enhanced RIG-I signaling, we determined whether DUSP11 deficiency would result in enhanced interferon expression during virus infection. NHDF cells with silenced *DUSP11* levels demonstrated approximately fourfold more induction of *IFNB1* mRNA levels upon M51R VSV infection (Supplemental Fig. S4A). We next determined if the reduction of RIG-I levels could rescue the virus replication defect in the cells lacking DUSP11. Knockdown of *RIG-I* in NHDF cells with reduced DUSP11 levels resulted in almost full rescue of replication of wild-type VSV (Fig. 4A; Supplemental Fig. S4B,C). Knocking down *RIG-I* also compensated for the virus replication deficits in A549 DUSP11 knockout cells infected with either M51R VSV (Fig. 4B; Supplemental Fig. S4D) or SINV (Fig. 4D). Furthermore, a similar trend was observed for infection of murine SVEC4-10 cells with M51R VSV (Fig. 4C; Supplemental Fig. S4E). These results demonstrate that the majority of the virus inhibitory effects of cells with lower DUSP11 expression are dependent on intact RIG-I signaling.

**Figure 5.**
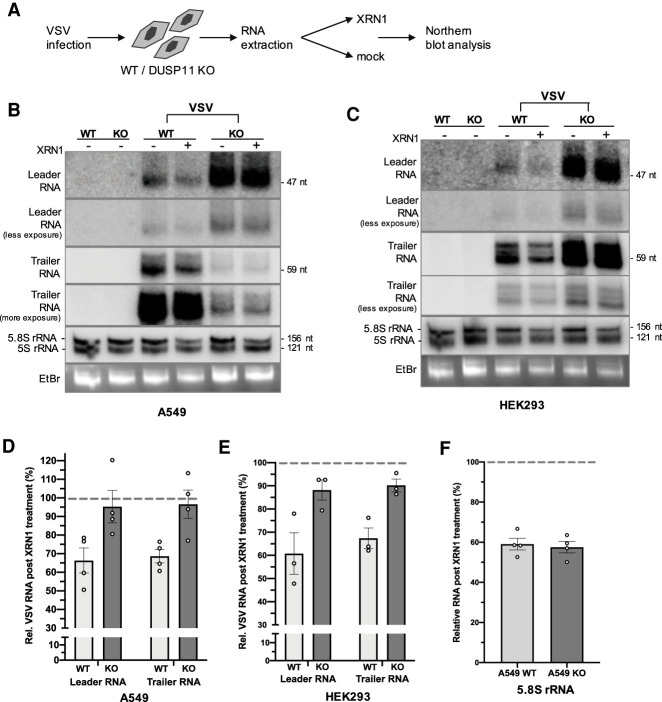
DUSP11 promotes RNA virus replication by dephosphorylating viral RNA PAMPs. (*A*) Schematic diagram of the VSV leader and trailer RNA 5′ end characterization assay. RNA was extracted from A549 or HEK293 WT and DUSP11 KO cells infected with VSV at MOI of five PFU/cell for 24 h and treated with or without 5′-monophosphate-dependent exonuclease XRN1. Purified RNA was then subject to Northern blot analysis. (*B*) Northern blot analysis on VSV leader, trailer and small RNAs purified from A549 WT/DUSP11 KO cells infected with WT VSV. (*C*) Northern blot analysis on VSV leader, trailer, and small RNAs purified from HEK293 WT/DUSP11 KO cells infected with WT VSV. (*D*) Graphical representation of the relative band density percentage ratio (+XRN1/−XRN1) of VSV leader and trailer RNA in A549 WT and DUSP11 KO cells as determined by Northern blot analysis in *B*. (*E*) Graphical representation of the relative band density percentage ratio (+XRN1/−XRN1) of VSV leader and trailer RNA in HEK293 WT and DUSP11 KO cells as determined by Northern blot analysis in *C*. (*F*) Graphical representation of the relative band density percentage ratio (+XRN1/−XRN1) of 5.8s rRNA in A549 WT and DUSP11 KO cells as determined by Northern blot analysis in *B*. Data are derived from *n* = 4 independent replicates for *D* and *F* and *n* = 3 independent replicates for *E*. In all panels, data are presented as mean ± SEM.

Because the DUSP11 catalytic activity promotes virus replication in a manner dependent on an intact RIG-I signaling pathway (Fig. 4A–D), we hypothesized that DUSP11 would directly modulate the 5′-triphosphate status of viral RNA transcripts. Therefore, we determined whether the viral PAMP RNAs known to activate RIG-I are targeted by DUSP11. VSV infection produces two small (47- and 59-nt) subgenomic 5′-triphosphate RNAs: leader RNA and trailer RNA (Colonno and Banerjee 1976; Schubert and Lazzarini 1981). Leader RNA is a known agonist of RIG-I (Plumet et al. 2007; Bitko et al. 2008; Oh et al. 2016). We infected A549 or HEK293 wild-type and DUSP11 knockout cells with wild-type VSV and conducted Northern blot analysis for leader or trailer RNA that was ex vivo treated with or without XRN1 (Fig. 5A; Supplemental Fig. S5). Because XRN1 treatment specifically degrades 5′-monophosphate but not 5′-triphosphate transcripts, if DUSP11 were acting directly on VSV transcripts, we would expect to observe a higher proportion of transcripts to be resistant to XRN1 in the DUSP11 knockout cells. Indeed, a higher proportion of leader and trailer RNAs from DUSP11 knockout cells was resistant to ex vivo 5′-monophosphate-specific XRN1-mediated turnover (Fig. 5B–E). Conversely, the control endogenous 5′-monophosphate 5.8S rRNA was equally susceptible to XRN1 regardless of DUSP11 status (Fig. 5F). Additionally, we observed that despite reduced replication of VSV, overall leader RNA levels accumulated to greater levels in both A549 and HEK293 DUSP11 knockout cells (Fig. 5B,C). In contrast to leader RNA, overall trailer RNA levels were reduced in A549 DUSP11 knockout cells (Fig. 5B). We speculate that this is due to trailer RNA accumulation being dependent on the full VSV replication cycle (Plumet et al. 2005, 2007), which is attenuated by the absence of DUSP11 in A549 but not HEK293 cell lines (Supplemental Fig. S3E). Combined, these results are consistent with small viral RNAs in DUSP11 knockout cells being resistant to nuclease-mediated turnover, similar to select host RNAP III transcripts that have been previously described as more triphosphorylated in the absence of DUSP11 (Burke et al. 2016).

We next explored the possibility that infection could alter DUSP11 biology. First, we examined the host RNAP III-transcribed vault RNAs and Y RNAs, previously shown to be higher in the absence of DUSP11 (Burke et al. 2016). Although a slight overall relative decrease was observed during infection, vault RNA1–3 (vtRNA1–3) and Y1 RNA levels remained higher in DUSP11 KO cells (Supplemental Fig. S6A). This suggests that infection did not dramatically alter DUSP11 activity associated with the turnover of host RNAP III transcripts (Burke et al. 2016; Belair et al. 2018). Previous studies demonstrated that in uninfected cells, DUSP11 is primarily localized in the nucleus (Yuan et al. 1998; Burke and Sullivan 2017). However, it is the cytosol where the life cycle of the majority of RNA viruses replicate and RIG-I sensing takes place. Therefore, we determined if virus infection altered DUSP11 subcellular localization. VSV infection of A549 cells consistently resulted in a modest decrease in overall steady-state and nuclear DUSP11 protein levels (∼40%–65% reduction) and a concurrent increase of cytosolic DUSP11 (∼3.2-fold higher than in the uninfected cytosol fraction, and an ∼5.2-fold higher cytoplasmic:nuclear ratio than in uninfected cells) (Supplemental Fig. S6B,C). Although DUSP11 remains overwhelmingly nuclear, the detection of cytosolic DUSP11 during infection is consistent with DUSP11 directly acting on viral transcripts localized in the cytosol. While these results do not rule out a contribution of host RNAP III RNAs to protection from virus infection in DUSP11 knockout cells, they do demonstrate that analogous to host-derived endogenous RNAs (Fig. 3E), viral transcripts known to activate RIG-I are modulated by DUSP11 during infection.

### Mice lacking DUSP11 display an enhanced signature of interferon signaling

**Figure 6.**
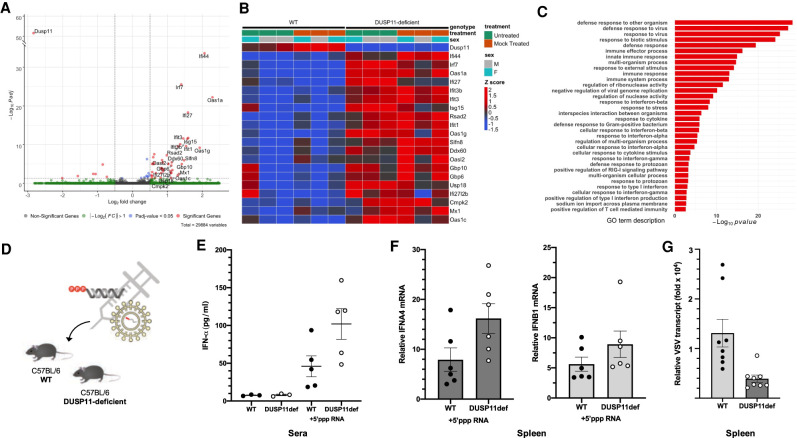
Mice lacking DUSP11 display an enhanced signature of interferon signaling. Transcriptomic analysis was performed on liver samples collected from age/sex pair-matched DUSP11-deficient (*n* = 6 mice: *n* = 3 male, *n* = 3 female) versus wild-type mice (*n* = 6 mice: *n* = 3 male, *n* = 3 female). Data were generated from two experiments: untreated (*n* = 3 mice: *n* = 2 male, *n* = 1 female) and mock-treated (*n* = 3 mice: *n* = 1 male, *n* = 2 female) conditions. Combined data were modeled controlling for sex and experiment-treatment (based on Supplemental Fig. S8A) using DESeq2. (*A*) Volcano plot of differentially expressed genes between DUSP11-deficient mice versus wild-type mice. (*B*) Heat map of differentially expressed genes up-regulated between DUSP11-deficient mice versus wild-type mice. (*C*) Analysis of gene ontology (GO) biological process terms enriched for genes up-regulated between DUSP11-deficient versus wild-type mice. (*D*) Schematic diagram of the VSV 5′-ppp PAMP RNA in vivo transfection assay and VSV infection assay. DUSP11-deficient (DUSP11def) and wild-type (WT) mice were either treated with in vitro transcribed VSV 5′-ppp PAMP RNA (intraperitoneal injection, 15 μg) or infected with WT VSV (intravenous tail vain injection, 2 × 10^6^ PFU). Sera and tissue samples were harvested 6 h posttreatment. (*E*) ELISA analysis of sera IFN-α levels from VSV 5′-ppp PAMP RNA or mock-treated mice. Sera were collected from age/sex pair-matched DUSP11-deficient (*n* = 3 mice: *n* = 1 male, *n* = 2 female for mock, and *n* = 5 mice: *n* = 3 male, *n* = 2 female for VSV 5′-ppp PAMP RNA-treated) and wild-type (*n* = 3 mice: *n* = 1 male, *n* = 2 female for mock, and *n* = 5 mice: *n* = 3 male, *n* = 2 female for VSV 5′-ppp PAMP RNA-treated) mice. (*F*) RT-qPCR analysis of IFNA4 and IFNB1 mRNA normalized to GAPDH mRNA in spleen RNA from VSV 5′-ppp PAMP RNA-treated mice. RT-qPCR results are presented relative to those of mock-treated mice. Spleen samples were collected from age/sex pair-matched DUSP11-deficient (*n* = 6 mice: *n* = 4 male, *n* = 2 female) and wild-type (*n* = 6 mice: *n* = 4 male, *n* = 2 female) mice. (*G*) RT-qPCR analysis of VSV transcript normalized to GAPDH mRNA in spleen RNA from VSV-infected mice. RT-qPCR results are presented relative to those of mock-treated uninfected mice. Spleen samples were collected from age/sex pair-matched DUSP11-deficient (*n* = 8 mice: *n* = 3 male, *n* = 5 female) and wild-type (*n* = 8 mice: *n* = 3 male, *n* = 5 female) mice. Data are presented as mean ± SEM.

Since our data indicate that DUSP11 has a conserved function of regulating the sensitivity of RIG-I induction in both human and mouse cell lines, we extended our studies to mice. We obtained DUSP11-deficient mice (*Dusp11^tm1a/tm1a^*) that contain an insertion directing enforced splicing of the *Dusp11* gene resulting in a premature stop codon (Supplemental Fig. S7). We collected tissue samples from age/sex pair-matched wild-type and DUSP11-deficient mice and conducted oligo-dT primed RNA sequencing on the liver and spleen. For the liver, two independent replicate experiments were analyzed under nonimmunostimulated conditions. Mice were either not treated (untreated) or treated with nonimmunostimulatory liposomal RNA (mock-treated) (Supplemental Fig. S8A), where mock-treated mice did not have a detectable immune response compared with untreated mice (Supplemental Fig. S8B,C). *Dusp11* transcript levels were sharply reduced in the DUSP11-deficient mice in all tissues tested (Fig. 6A; Supplemental Fig. S9D). Strikingly, transcriptome analysis of RNA from either untreated or mock-treated livers revealed increased expression of RIG-I-associated ISGs in the DUSP11-deficent mice (Fig. 6A,B; Supplemental Fig. S8D,E). Although type I interferon transcript levels were at the limit of detection, RT-qPCR analysis on select differentially regulated transcripts (*Oas1a* and *Ifi44* mRNA) independently confirmed elevated interferon and antiviral-response-related transcripts in the mock-treated liver samples from DUSP11-deficient mice (Supplemental Fig. S9A). The same trend was observed in mice possessing a different knockout allele of DUSP11 (*Dusp11^tm1b/tm1b^*, Supplemental Fig. S7), suggesting that the phenotype was not specific to a single transgenic line (Supplemental Fig. S9B). RT-qPCR analysis of these same ISGs (*Oas1a* and *Ifi44* mRNA) on RNA harvested from the kidney from mock-treated mice showed a similar albeit lower magnitude trend (Supplemental Fig. S9C). However, no apparent ISG transcriptional induction was observed in the spleen (Supplemental Fig. S9D). Despite a clear signature of enhanced interferon signaling in the liver and kidney, we did not detect enhanced interferon α (IFN-α) levels in the sera harvested from mock-treated DUSP11-deficient mice (Fig. 6E). Thus, enhanced interferon in DUSP11-deficient mice must be produced in a limited number of cells/cell types, or occur at such a low level so as to be difficult to detect. We conclude that, consistent with enhanced RIG-I signaling, reduced DUSP11 levels results in tissue-specific enhanced signatures of interferon signaling in vivo.

The results from our cell culture experiments predict that the enhanced interferon signature we detect in DUSP11-deficient mice is due to a reduced ability to process immunostimulatory triphosphorylated RNA. To test this, we used an established in vivo transfection method (Poeck et al. 2008) to deliver in vitro transcribed VSV 5′-triphosphate RNA (Goulet et al. 2013) into age/sex pair-matched wild-type and DUSP11-deficient mice (Fig. 6D; Supplemental Fig. S8A). Six hours postdelivery, enzyme-linked immunosorbent analysis (ELISA) on the sera revealed modestly enhanced (approximately twofold) IFN-α levels in the DUSP11-deficient mice (Fig. 6E). RT-qPCR analysis for *Ifna4* transcripts on RNA harvested from the spleen of these mice confirmed a similar trend (approximately twofold higher in DUSP11-deficient mice compared with wild-type mice) (Fig. 6F). Irrespective of DUSP11 status, the liver of all the 5′-triphosphate RNA-transfected mice displayed dramatically induced interferon-associated gene expression (Supplemental Fig. S10A–C), presumably due to the liver undergoing a high antiviral response from taking up a large share of the PAMP RNA injected into these mice. Nevertheless, under these conditions of high immune-stimulation, a few ISGs still retain higher expression in DUSP11-deficient mice, consistent with an altered antiviral response (Supplemental Fig. S10D). These findings demonstrate that DUSP11-deficient mice are more sensitive to triphosphorylated PAMP RNA.

To determine whether the increased interferon signaling signature we observed in DUSP11-deficient mice is associated with enhanced protection against viruses, we infected age/sex pair-matched wild-type and DUSP11-deficient mice with VSV (Fig. 6D). Consistent with the replication defects we observe in cultured cells (Fig. 4), DUSP11-deficient mice display reduced viral loads (∼3.3-fold reduction in viral transcript) in the spleen (Fig. 6G), on par with what has been reported for some other factors associated with the antiviral response (Wang et al. 2019; Zhu et al. 2019). Notably, we observed a greater magnitude of this effect in the female DUSP11-deficient mice (∼5.1-fold for females as compared with ∼1.6-fold for males) (Supplemental Fig. S11A). Additionally, we detected reduced *Ifna4* mRNA levels in infected DUSP11-deficient mice (∼3.5-fold) (Supplemental Fig. S11B). This is likely due to the reduced level of virus infection in these hosts, similar to what has been previously observed for other host modulators of the antiviral response (Wetzel et al. 2014; Liu et al. 2019). Combined, the above data demonstrate that DUSP11-deficient mice display an enhanced antiviral response and are modestly resistant to virus infection.

## Discussion

An emerging concept is that cells will use various mechanisms of manipulating nucleic acids to optimize the host response to infection. This includes altering the subcellular localization and posttranscriptional modifications of DNA or RNA. For example, mitochondrial DNA is released in the cytosol during infection with some RNA viruses (Aguirre et al. 2017). This results in an activation of host defenses previously associated with the PAMPs of DNA viruses (Ni et al. 2018). Other mitochondrial mechanisms have evolved to prevent leakage of mitochondrial RNA into the cytosol (Dhir et al. 2018). If these mechanisms are perturbed, this can trigger an inappropriate PRR MDA5 response. ADAR1-mediated posttranscriptional deamination of adenosine in structured and double-stranded RNAs prevents activation of several PRRs including MDA5, PKR, and RNase L (Rehwinkel and Gack 2020). These findings firmly establish that posttranscriptional modification is a key factor in a properly functioning innate immune response. Here we add control of the 5′-triphosphate status of RNA transcripts as a new mechanism for modulating PRR RIG-I sensitivity.

**Figure 7.**
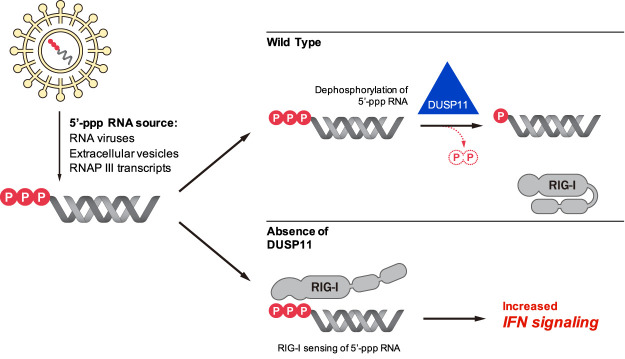
Model for DUSP11-mediated regulation of RIG-I signaling. The RNA triphosphatase DUSP11 dephosphorylates endogenous host and exogenous viral 5′-triphosphate RNAs and reduces the sensitivity of the RIG-I signaling response to these RNAs. The higher proportion of 5′-triphosphate RNAs in the absence of DUSP11 results in aberrant RIG-I sensing and increased interferon signaling.

Our results demonstrate that altering the levels of 5′-triphosphates on viral or host transcripts can increase or decrease their activation of RIG-I. We further show that a natural host mechanism, centered on DUSP11, modulates RNA triphosphate levels and associated proinflammatory activity. Experimental reduction of DUSP11 levels results in increased interferon signaling in the presence of RNA DAMPs/PAMPs. The ability of DUSP11 to alter interferon signaling is specific as it does not occur when primed with dephosphorylated RNAs or in the absence of RIG-I (Fig. 1D,E). Consistent with this, it was recently reported that lytic activation of Kaposi's sarcoma-associated herpesvirus (KSHV) reduces DUSP11 levels coincident with increased triphosphorylated RNAP III vault RNAs and enhanced MDA5 and RIG-I signaling (Zhao et al. 2018). A similar finding was observed during HIV infection, where reduced DUSP11 levels are observed coincident with an increase of host 5′-triphosphote Y RNAs and RIG-I signaling in infected cells (Vabret et al. 2019). Combined, these findings support the model that DUSP11 is part of a previously unknown surveillance system than can recognize and modify select host or viral 5′-triphosphate RNAs, altering their visibility to RIG-I (Fig. 7). This model identifies several targets for altering the cellular 5′-triphosphate balance, which may represent a fruitful area for exploring new ways to therapeutically harness the interferon response.

Recent work established the validity of activating PRRs as a strategy to enhance a therapeutic immune response against tumors. Agonists of STING and RIG-I have shown promise in mouse models as standalone or adjuvants for enhancing immunotherapies (Elion et al. 2018; Ramanjulu et al. 2018; Heidegger et al. 2019; Jiang et al. 2019). Conversely, in some contexts, interferon signaling is beneficial to tumor growth (Khodarev et al. 2012; Boelens et al. 2014; Nabet et al. 2017). Our work demonstrates that increasing or decreasing DUSP11 levels can alter the level of RIG-I activation that is triggered by coculture of tumor and fibroblast cells. These findings warrant further study into the possible applications of altering DUSP11 activity (increasing or decreasing) as a novel approach for manipulating tumor biology and the innate immune response to cancers.

Our current work combined with previous studies (Amador-Cañizares et al. 2018; Kincaid et al. 2018) demonstrates that DUSP11 can be either proviral or antiviral depending on the context. DUSP11 serves to promote biogenesis/activity of noncanonical retroviral and adenoviral microRNAs (Burke et al. 2016). We demonstrate here that DUSP11 activity is also advantageous to several different classes of RNA viruses via down-regulation of RIG-I signaling. However, DUSP11 activity serves an antiviral role in HCV infection (Amador-Cañizares et al. 2018; Kincaid et al. 2018). This apparent discrepancy can be explained by the sensitivity of HCV to XRN-mediated restriction and that virus's highly effective ability to counter the effects of RIG-I (Sedano and Sarnow 2014; Li et al. 2015; Thibault et al. 2015). Indeed, in the presence of miR-122, which protects HCV transcripts from XRN, reducing DUSP11 no longer conveys as strong an antiviral effect (Amador-Cañizares et al. 2018; Kincaid et al. 2018). Combined, these observations suggest that only those viruses that have evolved to allow their transcripts to engage the host triphosphate-tuning machinery will benefit from DUSP11.

Given that DUSP11 activity can modulate the sensitivity of RIG-I and associated interferon signaling, it stands to reason that there should be mechanisms to regulate DUSP11 activity. At this time, such mechanisms are unknown. We speculate that it would be advantageous for cells to decrease DUSP11 activity when a virus is detected and to increase activity when excessive triphosphorylated RNAs are encountered in uninfected cells. Along these lines, it remains possible that the reduced DUSP11 levels observed during KSHV and HIV infection (Zhao et al. 2018; Vabret et al. 2019) may be mediated in part by the host response to infection. In support of this possibility, we observe altered steady state levels and nuclear/cytoplasmic ratios during VSV infection (Supplemental Fig. S6B,C).

In uninfected contexts, cell types with high levels of DUSP11 activity could serve to safely remove the potentially immunostimulatory RNAP III-transcribed triphosphorylated RNA “waste products” that have been shown to be a major component of extracellular vesicle cargoes (Baglio et al. 2016; Shurtleff et al. 2017). If DUSP11 surveys and acts on incoming cargoes to remove 5′-triphosphates from RNA, it is possible that DUSP11-rich cell types exist that are proficient in clearing these proinflammatory waste products. An area worth further inquiry is determining which cell types have the most DUSP11 activity and precisely determining the location within the cell that DUSP11 encounters host and viral triphosphorylated RNAs. The ability of DUSP11 to act on incoming 5′-triphosphate EV RNAs and the cytoplasm-restricted HCV and VSV transcripts demonstrates activity within the cytosol, perhaps associated with endocytosis machinery or the extracellular membrane. Consistent with this, DUSP11 is localized in both the nucleus and cytosol (Supplemental Fig. S6B,C; Burke and Sullivan 2017; Amador-Cañizares et al. 2018) raising the possibility that DUSP11 functions in multiple cellular machines to regulate triphosphate levels.

Excessive induction of nucleic acid-triggered type I interferon is implicated in several autoinflammatory disorders collectively referred to as type I interferonopathies. The underlying interferon signaling signature observed in DUSP11-deficient mice (Fig. 6A–C; Supplemental Fig. S9A–C) is consistent with RIG-I-dependent type I interferon activation. However, the ISG transcriptional response of the DUSP11-deficient mice varied between tissues (Fig. 6; Supplemental Fig. S9). This is analogous to the tissue-specific type I interferon signature reported in models of several type I interferonopathies (Rehwinkel et al. 2013; Kim et al. 2016; Mackenzie et al. 2016). DUSP11-deficient mice displayed a twofold induction of serum IFN-α levels in response to 5′-triphosphate RIG-I agonists compared with wild-type mice (Fig. 6E). A similar level of modest type I interferon increase was observed in patients and mice harboring an activating genetic mutation in MDA5 (Gorman et al. 2017). Human peripheral blood mononuclear cells (PBMCs) from these patients stimulated with poly(I:C), a synthetic double-stranded RNA, exhibited a twofold induction of IFN-β and a subtle increase in ISG levels and a similar phenotype was observed in mice generated with an identical genetic mutation (Gorman et al. 2017). This subtle enhancement in basal type I interferon signaling not only granted MDA5 mutant mice significant protection against virus infection, but also promoted increased risk of autoinflammatory disease (Gorman et al. 2017). This suggests the possibility that DUSP11 could be involved in similar processes. Along these lines, it is interesting to note that a very recent study reports that DUSP11-deficient mice have a pathological enhanced LPS-triggered inflammatory response attributable to induced TAK1-mediated cytokine production (Yang et al. 2020). Although the authors attribute this phenotype to TAK-1 being a possible protein substrate of DUSP11, it is important to point out that enhanced RIG-I signaling, similar to what we report here for DUSP11-deficient cells/mice, is known to enhance TAK-1 phosphorylation (Mikkelsen et al. 2009; Maelfait and Beyaert 2012). Determining whether the type I interferon signature we observe in DUSP11-deficient mice is associated with enhanced increased inflammatory disease is a major goal of future studies.

In summary, we identified a previously unknown mechanism that regulates the sensitivity of the host response to immunostimulatory DAMP and PAMP transcripts centered on controlling RNA 5′-triphosphate levels. The challenges associated with triggering effective and timely immune responses without harming the host are substantial and necessarily involve numerous and overlapping mechanisms. In the case of structured triphosphorylated transcripts, at least part of the solution appears straightforward: enzymatic control of 5′-triphosphate levels by DUSP11.

## Materials and methods

### Mice

C57BL/6N Dusp11^tm1a(EUCOMM)Wtsi^ mice were generated by IVF using frozen sperm (EM: 09991) obtained from the European Mouse Mutant Archive and maintained on the C57BL/6N (Taconic) background. The knockout first (Skarnes et al. 2011) Dusp11^tm1a(EUCOMM)Wtsi^ mutation encodes a splicing acceptor (SA) following *Dusp11* exon 1 that disrupts targeted gene expression (Supplemental Fig. S7). The Dusp11^tm1a^ allele was converted to Dusp11^tm1b^ in mouse embryos in vitro using cell-permeable Cre recombinase (Excellgen EG-1001) (Ryder et al. 2014), which generates a full Dusp11-null allele without the *neomycin* cassette (Supplemental Fig. S7). Age-/sex-matched C57BL/6N wild-type and homozygote Dusp11^tm1a^ mice were used in all experiments except for Supplemental Fig. S9B where age-/sex-matched pairs of wild-type and the homozygote Dusp11^tm1b/tm1b^ mice were used. Embryo reconstitution and allele conversion were performed at the Mouse Genetic Engineering Facility (MGEF) at the University of Texas at Austin. All animal procedures were performed in compliance with the approved University of Texas at Austin Animal Care and Use Committee protocol. Detailed methods on mouse genotyping are described in the Supplemental Material.

### Cell lines and viruses

A549 and HEK293 DUSP11 CRISPR–Cas9 targeted cell lines were previously described (Burke et al. 2016). Detailed information on sources and conditions for all cell lines (MDA-MB-231, HFF, SVEC4-10, NHDF, Vero, and BHK-21) and viruses (VSV, Sindbis, and HSV-1) are described in the Supplemental Material.

### Viral infections and plaque assays

NHDF, A549, HEK293 or SVEC4-10 cells were plated in six-well or 12-well plates for virus infection. After 1 h of virus adsorption with gentle shaking at 15-min intervals, virus inoculum was aspirated from the wells and washed gently with PBS before adding growth medium. Two-hundred microliters of medium was collected at select time points postinfection and stored at −80°C to titer viral yield. Virus titer was quantified by standard plaque assay on BHK-21 or Vero cells in six-well plate format, serially diluted in a countable range (five to 250 plaques per well). Twenty-four hours (SINV) or 72 h (VSV) postcarboxymethyl cellulose (2%–3%) overlay, plates were fixed and stained with 0.25% Coomassie blue in 10% acetic acid, 45% methanol, or 0.5% methylene blue in 50% methanol.

### siRNA-mediated knockdown of DUSP11 and RIG-I

For coculture experiments, MDA-MB-231 and HFF cells were transfected with Silencer-negative control number 1 siRNA (Ambion AM4636), human DUSP11 siRNA (Ambion AM16708; siRNA ID: 105842) or human RIG-I (DDX58) siRNA (human; Ambion 4392420) using Lipofectamine RNAiMAX reagent (Thermo Fisher Scientific). For virus infection experiments, NHDF, A549, or SVEC4-10 cells were transfected with Silencer-negative control number 1 siRNA, human DUSP11 siRNA or human/mouse DDX58 siRNA (mouse; Ambion 4390771). Detailed siRNA conditions are described in the Supplemental Material.

### Extracellular vesicle isolation

MDA-MB-231 and HFF cells cocultured for EV production were grown in EV-free medium produced by pelleting EVs by centrifuging 2× DMEM growth medium (20% FBS, 2% penicillin and streptomycin) at 100,000*g* in a SW-28 rotor (Beckman Coulter) for 24 h at 4°C and collecting the EV-cleared supernatants. EV-free growth medium was diluted to 1× with serum free medium. EVs were isolated from MDA-MB-231 and HFF coculture conditioned medium by differential centrifugation as previously described (Théry et al. 2006; Shurtleff et al. 2017). Detailed methods are described in the Supplemental Material.

### In vitro RNA transcription

In vitro transcription of the HCV-5′ UTR and VSV 5′-triphosphate RNA was prepared as previously described (Goulet et al. 2013; Kincaid et al. 2018). In vitro transcribed RNA was synthesized using the AmpliScribe T7-Flash (Epicentre) transcription kit following the manufacturer's instructions.

### RNA phosphatase treatment

In vitro transcribed HCV 5′ UTR RNA was treated with calf intestine phosphatase (CIP; New England Biolabs) and in vitro translated DUSP11 core protein in conditions previously described (Kincaid et al. 2018). TRIzol reagent was added to terminate the reaction and retrieve the RNA.

### 5′ end characterization assay

5′–3′ exonuclease XRN1 (New England Biolabs) or Terminator (Epicentre) was used to characterize the 5′ end of coculture EV RNA and VSV RNA as previously described (Burke et al. 2014). Detailed methods are described in the Supplemental Material.

### In vivo administration of 5′-triphosphate RNA and VSV

In vitro transcribed VSV 5′-triphosphate PAMP RNA was complexed with in vivo JetPEI (PolyPlus) using an N/P (nitrogen/phosphate) ratio of 8. Each batch of in vitro transcribed PAMP RNA was screened in cell culture to ascertain its ability to elicit an interferon response (Supplemental Fig. S8C). RNA lacking immunostimulatory activity was used in the mock transfected mice. No transfection reagent was used in the untransfected mice. Treated mice were administered with 15 μg of RNA through the intraperitoneal (IP) route. For VSV infection, mice were infected with wild-type VSV Indiana strain (2 × 10^6^ PFU) via intravenous tail vein injection. Tissue samples and sera were collected 6 h postadministration. Harvested tissue samples were immediately transferred to lysing matrix tubes (Lysing Matrix D, MP Biomedicals) containing TRIzol and were homogenized in a homogenizer (Bead Mill, Fisher Scientific) before snap-freezing in liquid nitrogen.

### Northern blot analysis

Northern blot analysis was performed as previously described (oligo probe sequence information in Supplemental Table S1; McClure et al. 2011). Uncropped Northern blots are presented in Supplemental Figure S13. Detailed methods are described in the Supplemental Material.

### Real-time quantitative PCR

Total RNA was extracted from cells using TRIzol reagent or PIG-B. Snap-frozen tissue samples were thawed and homogenized in the lysing matrix tubes containing TRIzol prior to RNA extraction. Gene expression of human or mouse DUSP11, IFNB1, IFNA4, ISG15, MX1, OAS1a, DDX58 (RIG-I), IFI44, IL-6, and IFIT1 was normalized to the expression of GAPDH; 7SL, vtRNA1-3, Y1, and 5.8S RNA expression was normalized to 5S rRNA (primer sequence information in Supplemental Table S1). Detailed conditions are described in the Supplemental Material.

### Immunoblot analysis

Protein was extracted from cells lysed in RIPA buffer or SDS lysis buffer and cell lysates were fractionated on SDS-PAGE. Uncropped Northern blots are presented in Supplemental Figure S12. Detailed methods are described in the Supplemental Material.

### Enzyme-linked immunosorbent assay

Whole blood from age-/sex-matched C57BL/6N wild-type and homozygote *Dusp11^tm1a/tm1a^* mice were collected 6 h postadministration of in vitro transcribed VSV 5′-triphosphate RNA. Whole blood samples were left for 15 min at room temperature followed by centrifugation at 2000*g* for 10 min at 4°C to separate the serum from the whole-blood samples. IFN-α levels were detected using the VeriKine-HS mouse IFN α all subtype ELISA kit (PBL 42115-1) according to the manufacturer's instructions.

### RNA-seq library preparation and data analysis

Libraries were prepared using RNA purified from mouse tissue samples for the BGISEQ-500 DNBseq platform (BGI). Briefly, mRNAs were isolated using oligo(dT)-attached magnetic beads, fragmented and reverse-transcribed for cDNA synthesis. Adapter-ligated cDNA fragments were PCR amplified and libraries were validated using the Agilent 2100 Bioanalyzer. Qualified double-stranded PCR products were then circularized to produce the final single-strand circle DNA library. Libraries were amplified with φ29 to generate DNA nanoballs and were loaded onto flow cells for 100-bp paired-end sequencing. Detailed data analysis methods are described in the Supplemental Material.

### Statistical analysis

Graphpad Prism software was used for statistical analyses. Experimental groups were compared using the two-tailed Students *t*-test to determine *P*-values. *P* < 0.05 were considered statistically significant and significance was shown by the presence of asterisks above data points with one or two asterisks representing *P* < 0.05 or *P* < 0.01, respectively. *P*-values > 0.05 are indicated as the actual number on the graph. Error bars were presented as mean ± standard error of the mean. Statistical consultation was provided by the Department of Statistics and Data Sciences at the University of Texas at Austin.

### Data availability

All data sets described in Supplemental Table S2 are available on the GEO database (GSE158837). All primary source data reported in this study are archived in the Mendeley data repository (DOI: 10.17632/5jvrt3k2t6.1).

## Supplementary Material

Supplemental Material
