# Supplementary material for: DUSP11-mediated control of 5′-triphosphate RNA regulates RIG-I sensitivity
Source: Genes Dev. 2020 Dec 1;34(23-24):1697–712. doi: 10.1101/gad.340604.120 (PMC7706711; doi:10.1101/gad.340604.120)
Supplement: Supplemental Material [file supp_34_23-24_1697__index.html]

DUSP11-mediated control of 5′-triphosphate RNA regulates RIG-I sensitivity — Supplemental Material 

# DUSP11-mediated control of 5′-triphosphate RNA regulates RIG-I sensitivity

## Supplemental Material

- Supplemental\_Material.pdf
