# Supplementary material for: DUSP11-mediated control of 5′-triphosphate RNA regulates RIG-I sensitivity
Source: Genes Dev. 2020 Dec 1;34(23-24):1697–712. doi: 10.1101/gad.340604.120 (PMC7706711; doi:10.1101/gad.340604.120)
Supplement: Supplemental Material [file supp_gad.340604.120_Supplemental_Material.pdf]

Supplemental Material

DUSP11-mediated Control of 5'-Triphosphate RNA Regulates RIG-I Sensitivity

Authors

Joon H. Choi[1], James M. Burke[1,2], Kayla H. Szymanik[1], Upasana Nepal[1], Anna Battenhouse[1], Justin T. Lau[1], Aaron Stark[1], Victor Lam[1,3], Christopher S. Sullivan[1]*

Affiliations

[1]Department of Molecular Biosciences, The University of Texas at Austin, 1 University Station A5000, Austin TX 78712-0162

[2]Current Affiliation: Department of Biochemistry, University of Colorado, Boulder, CO 80309, USA.

[3]Current Affiliation: Department of Pharmaceutical Chemistry, University of California, San Francisco, CA 94158

*Corresponding author: chris_sullivan@austin.utexas.edu

Supplemental Material includes:

Supplemental Figures: S1-S13

Supplemental Tables: S1-S2

Supplemental materials and methods

Supplemental references

Supplemental Figures

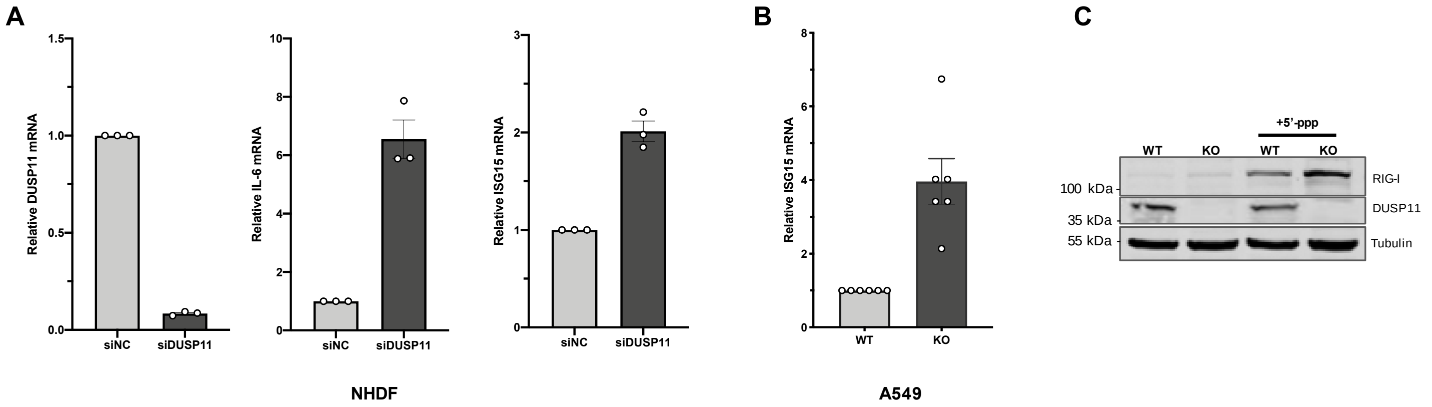

Supplemental Fig. S1. Induction of interferon response associated genes in DUSP11-deficient cells. (A) RT-qPCR analysis of DUSP11, IL-6 and ISG15 mRNA levels normalized to GAPDH mRNA in NHDF cells transfected with siRNA against DUSP11 (siDUSP11) relative to cells transfected with the negative control siRNA (siNC). (B) RT-qPCR analysis of ISG15 mRNA levels normalized to GAPDH mRNA in A549 DUSP11 knockout (KO) cells relative to wild-type (WT) cells. Note, these data points are an alternatively plotted version (identical data) as plotted in Fig. 1B, D, and were derived from cells mock-treated with Lipofectamine. (C) Immunoblot analysis assessing RIG-I and DUSP11 expression in A549 WT and DUSP11 KO cells transfected with 15 ng of *in vitro* transcribed 5'-ppp RNA for 18 hours. Data are derived from n=3 independent replicates for (A) and n=6 independent replicates for (B). Data are presented as mean ± SEM.

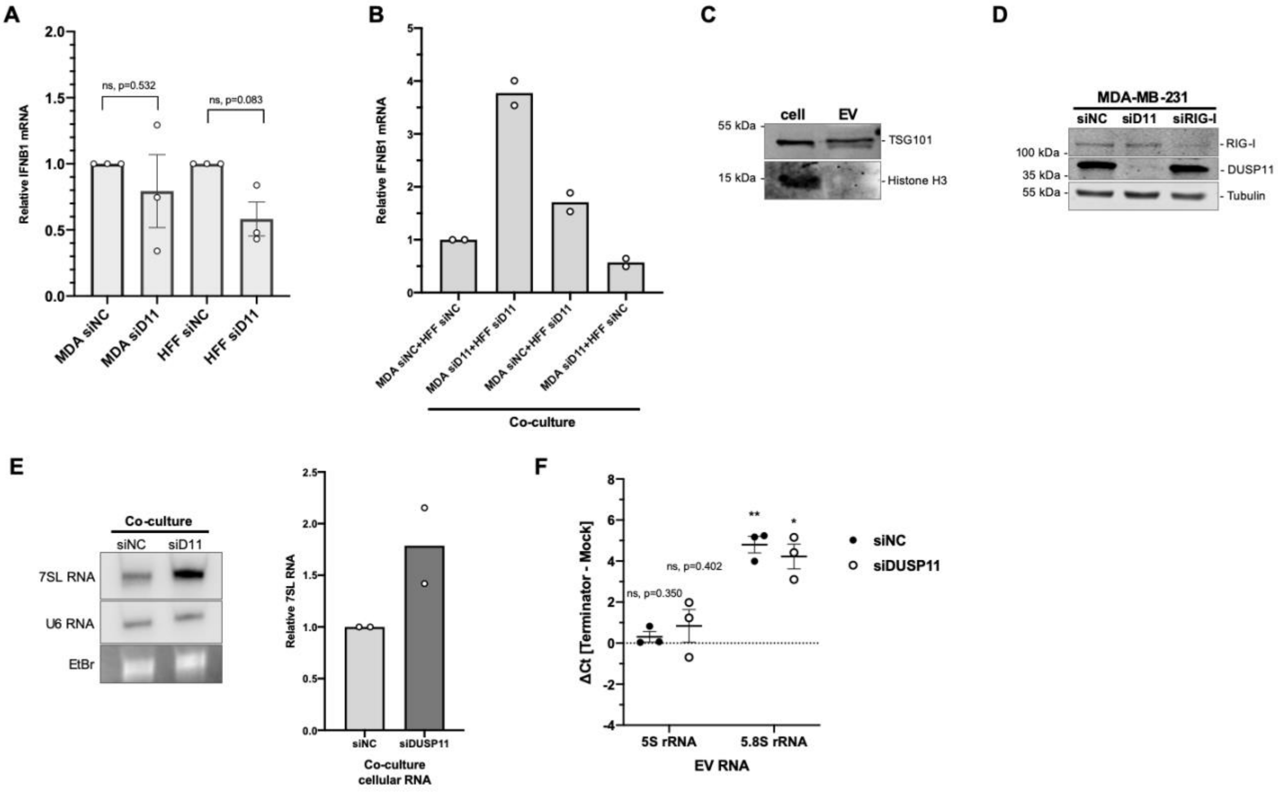

Supplemental Fig. S2. Co-culture of fibroblast and tumor cells. (A) RT-qPCR analysis of IFNB1 mRNA transcript levels in MDA-MB-231 (MDA) and HFF cells transfected with siRNAs against DUSP11 (siD11) relative to cells transfected with the negative control siRNA (siNC). (B) RT-qPCR analysis of IFNB1 mRNA transcript levels in MDA-MB-231 and HFF cells individually treated with either siNC or siDUSP11 followed by co-culture. Results are relative to co-cultured MDA-MB-231 siNC and HFF siNC cells. (C) Immunoblot analysis of whole cell lysate and EV-enriched preparation to assess enrichment of extracellular vesicles. EV marker TSG101 and the nuclear protein histone H3 are shown. Whole cell lysate and conditioned media were collected from MDA-MB-231 and HFF co-cultured cells. EV protein was isolated from co-culture conditioned media using differential centrifugation. (D) Immunoblot analysis assessing siRNA knockdown of DUSP11 and RIG-I in MDA-MB-231 cells. (E) Northern blot analysis (left) on cellular 7SL RNA and U6 RNA loading control purified from co-cultured cells silenced for DUSP11 (siD11) or negative control (siNC) and graphical representation (right) of the band density of 7SL RNA normalized to U6 RNA relative to RNA from siNC co-culture. (F) RT-qPCR analysis of 5S rRNA and 5.8S rRNA with or without Terminator treatment on RNA isolated from EVs. EVs were derived from conditioned media of co-cultured cells treated with siNC or siDUSP11. ΔCt values were calculated by subtracting mock-treated from Terminator-treated EV RNA Ct values. Data are derived from n=3 independent replicates for (A, F) and n=2 independent replicates for (B, E). Data are presented as mean ± SEM. *P<0.05, **P<0.01 (two-tailed Student's t-test).

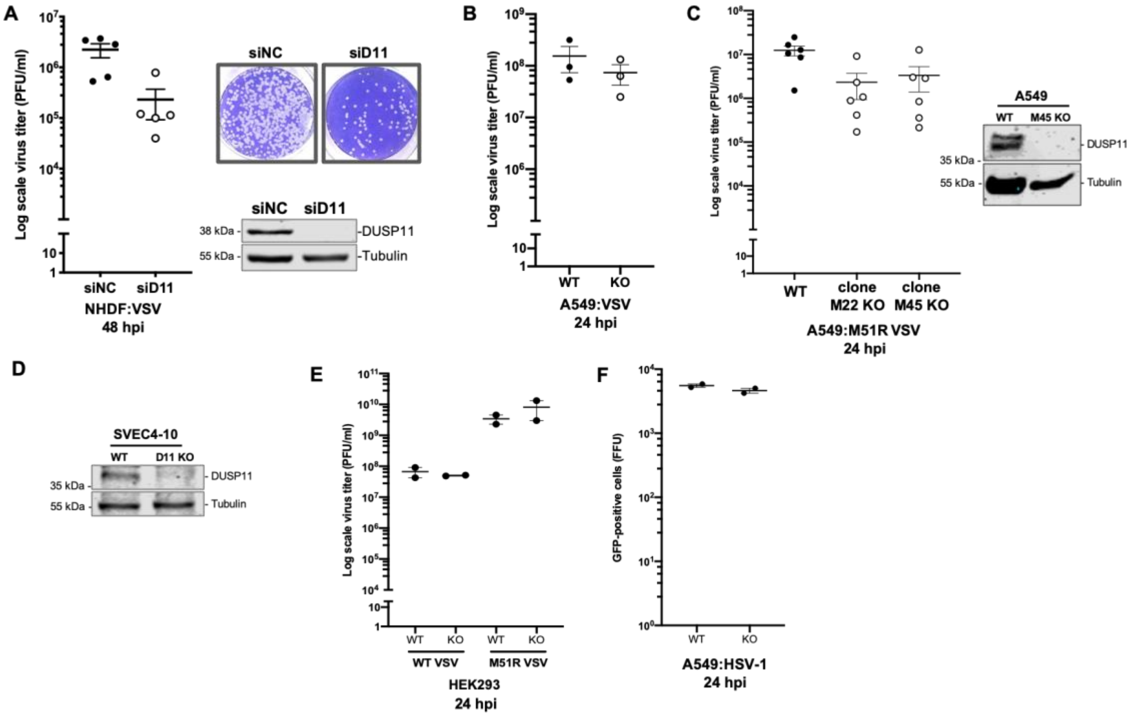

Supplemental Fig. S3. Virus replication in cells lacking DUSP11 expression. (A) VSV viral titer of negative control siRNA (siNC) and siRNA targeting DUSP11 (siDUSP11)-treated NHDF cells determined by plaque assay (left), representative image of plaque assay (top right), and immunoblot analysis (bottom right) to assess siRNA knockdown of DUSP11 in NHDF cells. Cells treated with siNC or siDUSP11 were infected with WT VSV at MOI of 0.1 PFU/cell and virus supernatant was collected 48 hours post infection (hpi) for plaque assay analysis. (B) VSV titer on viruses obtained from A549 wild-type (WT) or DUSP11 knockout (KO) cells as determined by plaque assay. Cells were infected with WT VSV at MOI of 0.05 PFU/cell and virus supernatant was collected 24 hpi. (C) M51R VSV viral titer of A549 WT and DUSP11 KO clone M22 used throughout this study as well as an independent clone, M45, was determined by plaque assay. Cells were infected with M51R VSV at MOI of 0.05 PFU/cell and virus supernatant was collected 24 hpi for plaque assay analysis. Immunoblot analysis assessing DUSP11 expression in A549 WT and DUSP11 knockout clone M45. (D) Immunoblot analysis assessing DUSP11 expression in SVEC4-10 WT and DUSP11 KO cells. (E) VSV titer on viruses obtained from HEK293 WT or DUSP11 KO cells as determined by plaque assay. Cells were infected with WT or M51R VSV at MOI of 0.05 PFU/cell and virus supernatant was collected 24 hpi. (F) HSV-1(17+)-BAC-eGFP viral titer on viruses obtained from A549 WT or DUSP11 KO cells as determined by FFU. Cells were infected with HSV-1 at MOI of 0.05 PFU/cell and virus supernatant was collected 24 hpi. Data are derived from n=5 independent replicates in (A), n=3 independent replicates in (B), n=6 independent replicates in (C), and n=2 independent replicates in (E, F). Data presented as mean ± SEM.

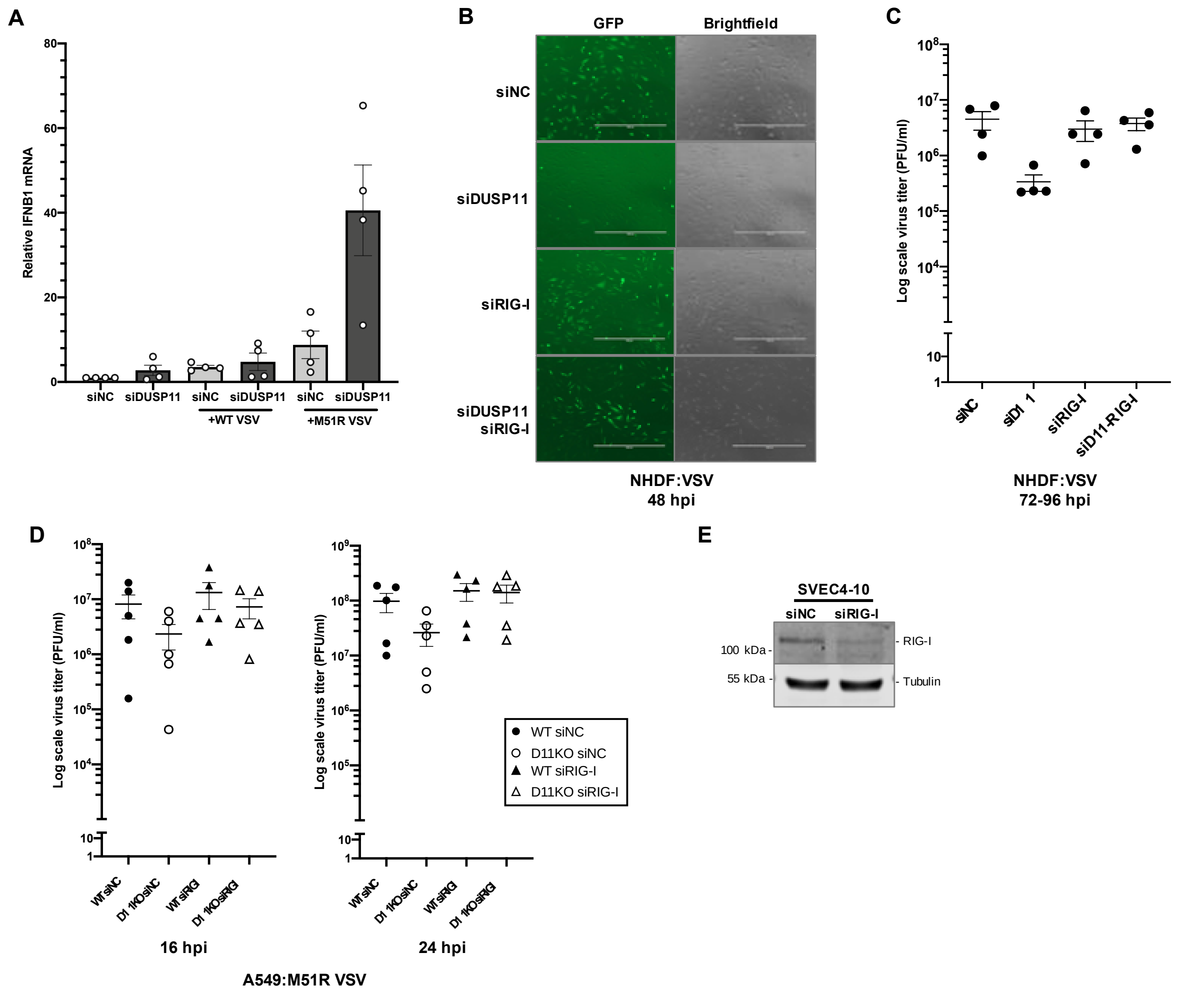

Supplemental Fig. S4. Silencing of RIG-I expression recovers deficit in virus replication in DUSP11 knockdown cells. (A) RT-qPCR analysis of IFNB1 mRNA transcript levels in NHDF cells treated with negative control siRNA (siNC) or siRNA targeting DUSP11 (siDUSP11) and infected with WT VSV or M51R VSV at MOI of 1 PFU/cell for 6 hpi. Results are relative to mock-treated siNC-treated cells. (B) Representative GFP and brightfield image of virus infection from Fig. 4A. Images were taken 48 hours post infection (hpi). (C) VSV viral titer of negative control siRNA (siNC), siRNA targeting DUSP11 (siD11) or RIG-I (siRIG-I)-treated NHDF cells determined by plaque assay. Cells treated with siRNA were infected with WT VSV at MOI of 0.25 PFU/cell and virus supernatant was collected 72-96 hpi for plaque assay analysis. (D) M51R VSV viral titer of siNC or siRIG-I-treated A549 WT and DUSP11 KO cells determined by plaque. Cells treated with siRNA were infected with M51R VSV at MOI of 0.05 PFU/cell and virus supernatant was collected 16 (left) and 24 (right) hpi for plaque assay analysis. (E) Immunoblot analysis assessing siRNA knockdown of RIG-I in SVEC4-10. Data are derived from n=4 independent replicates for (A, C) and n=5 independent replicates for (D). Data are presented as mean ± SEM.

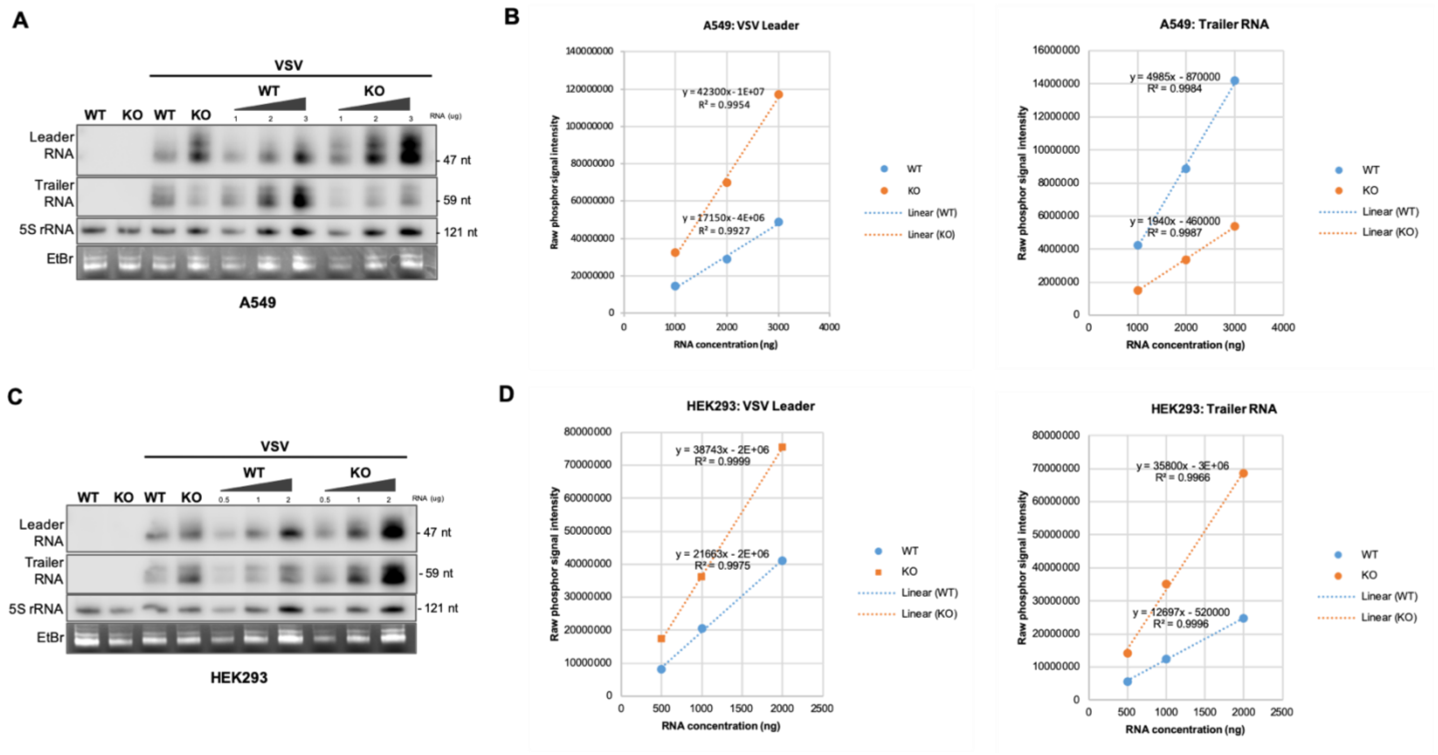

Supplemental Fig. S5. Linearity of VSV leader and trailer RNA expression as determined by northern blot analysis in WT and DUSP11 KO cells. (A) Northern blot analysis on VSV leader and trailer RNA verifies that the VSV RNA levels, as determined by phosphor imaging analysis in Fig. 5. B, D are within the linear range. Total RNA was isolated from infected cells and northern blots analysis was conducted, loading three different amounts of RNA, spanning the amounts used in Fig. 5B. (B) Scatter plot of RNA dilution series collected from A549 WT and DUSP11 KO cells infected with WT VSV and corresponding raw phosphor signal intensity of VSV leader and trailer RNA from northern blot analysis in (A). (C, D) Analysis of northern blot linearity from infections conducted in HEK293, conducted as described above for A549 cells, demonstrates that the values determined for Fig. 5. C, E are within the linear range. For panels (B, D), the calculated R^2 values indicate a good fit to the linear model.

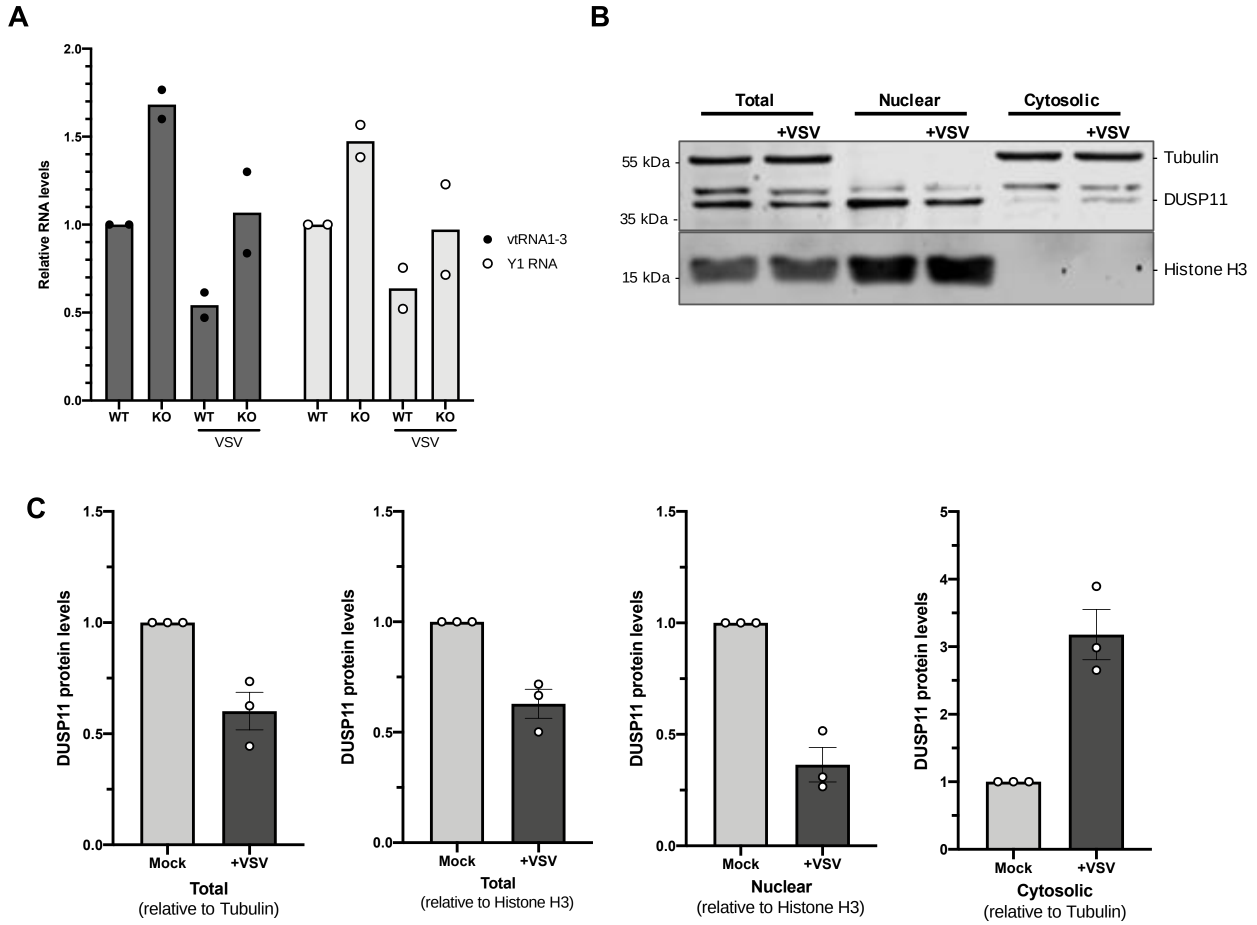

Supplemental Fig. S6. DUSP11 expression and localization during VSV infection. (A) RT-qPCR analysis of vault RNA1-3 (vtRNA1-3) and Y1 RNA levels normalized to 5S rRNA in A549 WT and DUSP11 KO cells infected with M51R VSV at MOI of 0.05 PFU/cell for 18 hpi. Results are relative to mock-treated WT cells. (B) Immunoblot analysis assessing DUSP11, Histone H3 (nuclear marker) and Tubulin (cytosolic marker) expression in whole cell and fractionated lysates from A549 WT and DUSP11 KO cells infected with WT VSV at MOI 0.05 PFU/cell for 18 hpi. (C) Graphical representation of the band density of DUSP11 protein in A549 cells infected with VSV relative to mock treated cells as determined by immunoblot analysis in (B). Total protein fractions were normalized to Tubulin or Histone H3, nuclear protein fraction to Histone H3, and cytosolic protein fraction to Tubulin. Data are derived from n=2 independent replicates for (A) and n=3 independent replicates for (C). Data presented as mean ± SEM.

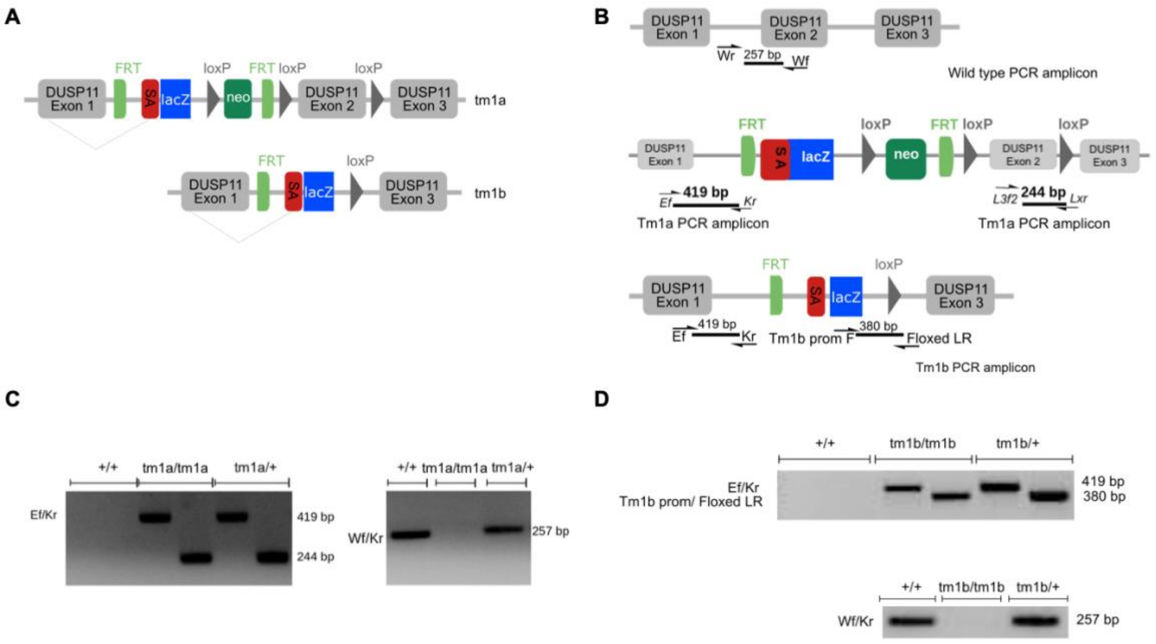

Supplemental Fig. S7. Genotyping strategy of Tm1a DUSP11-deficient and Tm1b DUSP11 knockout mice. (A) Diagram of knock-out first *Dusp11*^{tm1a} allele (top) and null *Dusp11*^{tm1b} allele (bottom). *Dusp11*^{tm1a} allele contains a splicing acceptor (SA) to disrupt targeted gene expression of *Dusp11* and *Dusp11*^{tm1b} is generated by deletion of exon 2 and the neomycin (neo) cassette using Cre recombinase. (B) Diagram of PCR genotyping strategy. The Wf/Wr primer set amplifies a 257 bp PCR amplicon from the wild-type genome (top). The Ef/Kr, L3f2/Lxr primer sets amplify a 419 bp and 244 bp PCR product respectively from the Tm1a allele (middle) and the Tm1b prom F/Floxed R primer set amplifies a 380 bp PCR product from the Tm1b allele (bottom). (C) Representative PCR genotyping results confirming genotype of wild-type (+/+), homozygote *Dusp11*$^{tm1a/tm1a}$ (tm1a/tm1a), and heterozygote *Dusp11*$^{tm1a/+}$ (tm1a/+) genomic DNA. (D) Representative PCR genotyping results confirming genotype of wild-type (+/+), homozygote *Dusp11*$^{tm1b/tm1b}$ (tm1b/tm1b), and heterozygote *Dusp11*$^{tm1b/+}$ (tm1b/+) genomic DNA.

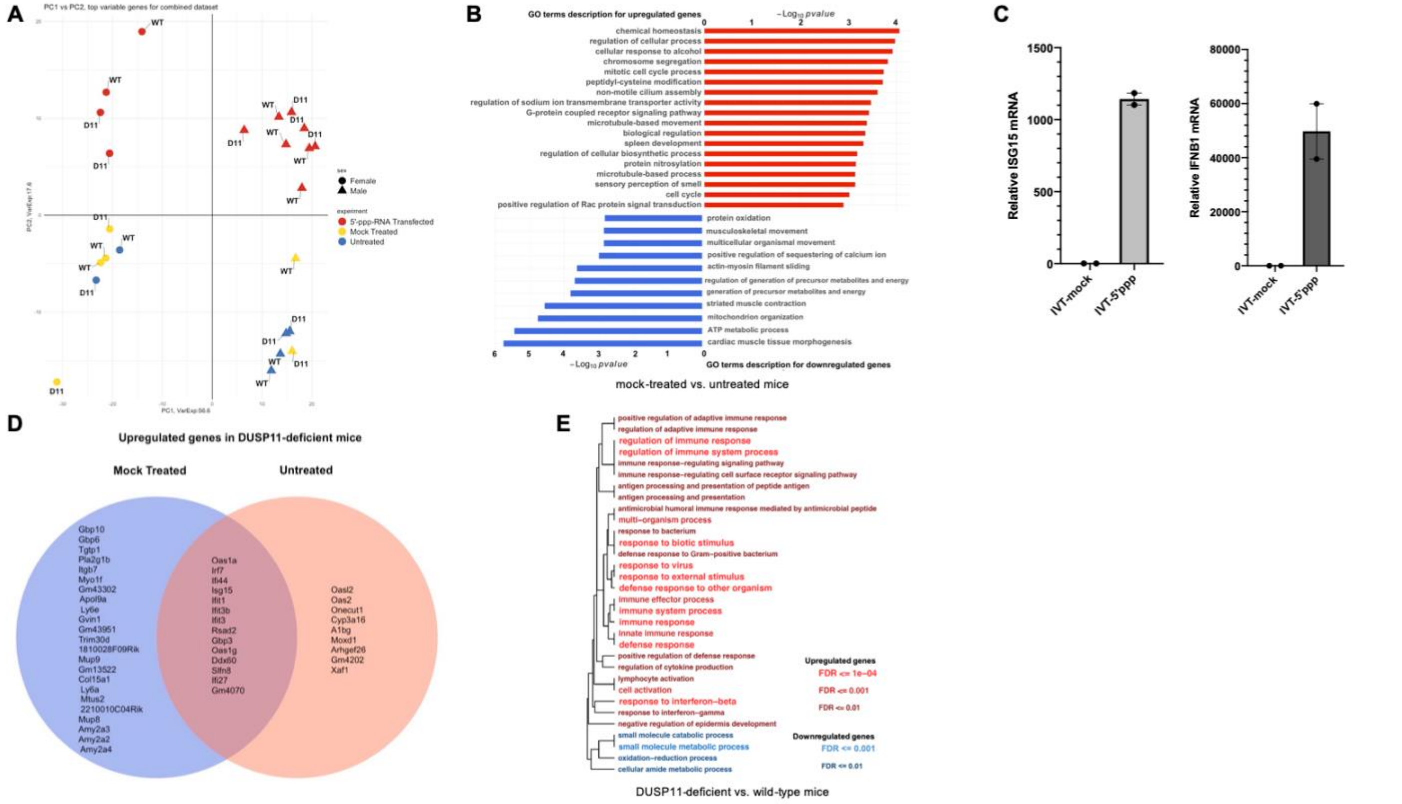
A
PC1 vs PC2, top variable genes for combined dataset
sex
Female
Male
experiment
5'-ppp-RNA Transfected
Mock Treated
Untreated
WT
D11
B
GO terms description for upregulated genes
chemical homeostasis
regulation of cellular process
cellular response to alcohol
chromosome segregation
mitotic cell cycle process
peptidyl-cysteine modification
non-motile cilium assembly
regulation of sodium ion transmembrane transporter activity
G-protein coupled receptor signaling pathway
microtubule-based movement
biological regulation
spleen development
regulation of cellular biosynthetic process
protein nitrosylation
microtubule-based process
sensory perception of smell
cell cycle
positive regulation of Rac protein signal transduction
protein oxidation
musculoskeletal movement
multicellular organismal movement
positive regulation of sequestering of calcium ion
actin-myosin filament sliding
regulation of generation of precursor metabolites and energy
generation of precursor metabolites and energy
striated muscle contraction
mitochondrion organization
ATP metabolic process
cardiac muscle tissue morphogenesis
GO terms description for downregulated genes
mock-treated vs. untreated mice
C
Relative ISG15 mRNA
Relative IFNB1 mRNA
IVT-mock
IVT-5'ppp
D
Upregulated genes in DUSP11-deficient mice
Mock Treated
Untreated
Gbp10
Gbp6
Tgtp1
Pla2g1b
Itgb7
Myo1f
Gm43302
Apol9a
Ly6e
Gvin1
Gm43951
Trim30d
1810028F09Rik
Mup9
Gm13522
Col15a1
Ly6a
Mtus2
2210010C04Rik
Mup8
Amy2a3
Amy2a2
Amy2a4
Oas1a
Irf7
Ifi44
Isg15
Ifit1
Ifit3b
Ifit3
Rsad2
Gbp3
Oas1g
Ddx60
Slfn8
Ifi27
Gm4070
Oasl2
Oas2
Onecut1
Cyp3a16
A1bg
Moxd1
Arhgef26
Gm4202
Xaf1
E
positive regulation of adaptive immune response
regulation of adaptive immune response
regulation of immune response
regulation of immune system process
immune response-regulating signaling pathway
immune response-regulating cell surface receptor signaling pathway
antigen processing and presentation of peptide antigen
antigen processing and presentation
antimicrobial humoral immune response mediated by antimicrobial peptide
multi-organism process
response to bacterium
response to biotic stimulus
defense response to Gram-positive bacterium
response to virus
response to external stimulus
defense response to other organism
immune effector process
immune system process
immune response
innate immune response
defense response
positive regulation of defense response
regulation of cytokine production
lymphocyte activation
cell activation
response to interferon-beta
response to interferon-gamma
negative regulation of epidermis development
small molecule catabolic process
small molecule metabolic process
oxidation-reduction process
cellular amide metabolic process
Upregulated genes
FDR <= 1e-04
FDR <= 0.001
FDR <= 0.01
Downregulated genes
FDR <= 0.001
FDR <= 0.01
DUSP11-deficient vs. wild-type mice

Supplemental Fig. S8. Transcriptome-wide analysis of the liver from untreated and mock-treated mice. (A) Principal component analysis (PCA) of liver RNA from untreated, mock-treated, and 5'-ppp-RNA-treated DUSP11-deficient and wild-type mice. Liver RNA were collected from age/sex pair-matched wild-type (n=3 mice: n=2 male, n=1 female for untreated, and n=3 mice: n=1 male, n=2 female for mock-treated, and n=6 mice: n=4 male, n=2 female for 5'-ppp-RNA-transfected) and DUSP11-deficient mice (n=3 mice: n=2 male, n=1 female for untreated, and n=3 mice: n=1 male, n=2 female for mock-treated, and n=6 mice: n=4 male, n=2 female for 5'-ppp-RNA-transfected). To reduce the main source of variance determined by PCA (sex, experiment-treatment), model terms for sex, treatment and genotype were run on data subsets in Fig. 6A-C. (B) Gene Ontology (GO) analysis of upregulated and downregulated differentially expressed genes between the mock-treated versus untreated analyses. (C) RT-qPCR analysis of ISG15 and IFNB1 mRNA normalized to GAPDH mRNA in *in vitro* transcribed mock RNA (IVT-mock) and VSV 5'-ppp PAMP RNA (IVT-5'ppp) transfected in A549 cells to verify immunogenicity of RNA. A549 cells in 12-well format were transfected with 100-250 ng of *in vitro* transcribed mock RNA or VSV 5'-ppp PAMP RNA and RNA lysates were collected for RT-qPCR analysis 24 hours post transfection. Results are presented relative to control cells treated with only Lipofectamine 2000. (D) Venn diagram of differential expressed genes in DUSP11-deficient mice between mock-treated versus untreated analyses. (E) Gene Ontology Mann-Whitney U (GO MWU) analysis of clusters of related Biological Process GO terms enriched in differentially expressed genes that are upregulated (red) and downregulated (blue) in DUSP11-deficient versus wild-type mice, using data presented in Fig. 6A-C. The color/size of the font indicates the False Discovery Rate (FDR) of the term as indicated in the insert key. For (C), data are derived from n=2 independent replicates and presented as mean ± SEM.

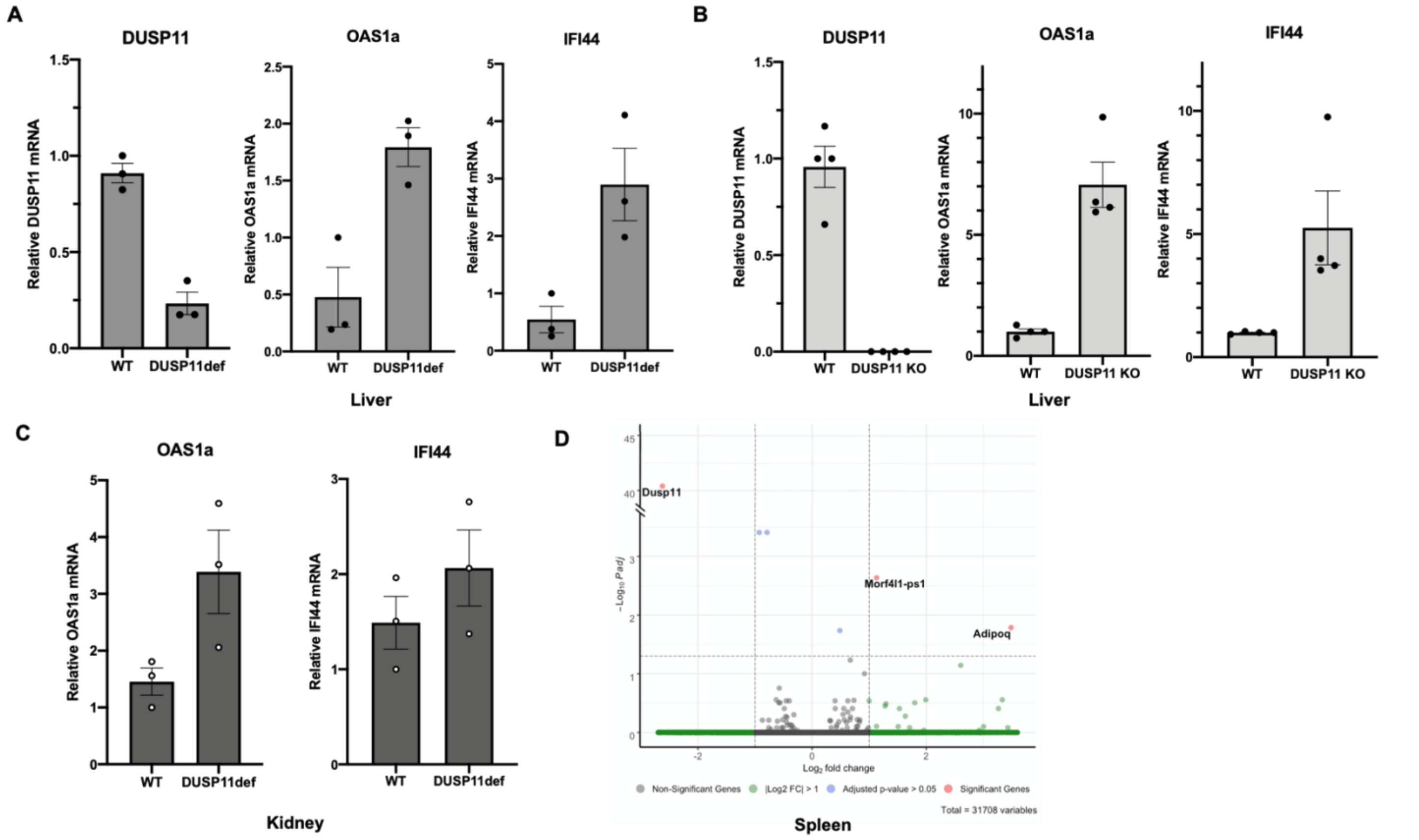

Supplemental Fig. S9. Tissue-specific interferon signaling signature in DUSP11-deficient (*Dusp11*$^{tm1a/tm1a}$) and DUSP11 KO (*Dusp11*$^{tm1b/tm1b}$) mice. (**A**) RT-qPCR analysis of DUSP11, OAS1a and IFI44 mRNA normalized to GAPDH mRNA in liver RNA from DUSP11-deficient *Dusp11*$^{tm1a/tm1a}$ (DUSP11def) and wild-type (WT) mice. RT-qPCR results are presented relative to one arbitrarily selected WT mouse. (B) RT-qPCR analysis of DUSP11, OAS1a and IFI44 mRNA normalized to GAPDH mRNA in liver RNA from a separate DUSP11 knockout allele, *Dusp11*$^{tm1b/tm1b}$ (DUSP11 KO) and wild-type (WT) mice. RT-qPCR results are presented relative to one arbitrarily selected WT mouse. (C) RT-qPCR analysis of OAS1a and IFI44 mRNA normalized to GAPDH mRNA in kidney RNA from wild-type and DUSP11-deficient mice. RT-qPCR results are presented relative to one arbitrarily selected WT mouse. (D) Volcano plot of differentially expressed genes in the spleen between DUSP11-deficient versus wild-type mice. For panels (A, C, D), tissue samples were collected from mock-treated age/sex pair-matched DUSP11-deficient (n=3 mice: n=1 male, n=2 female) and wild-type (n=3 mice: n=1 male, n=2 female) mice. For (B), tissues samples were collected from age/sex pair-matched DUSP11 KO (n=4 mice: n=2 male, n=2 female) and wild-type (n=4 mice: n=2 male, n=2 female) mice. Data are presented as mean ± SEM.

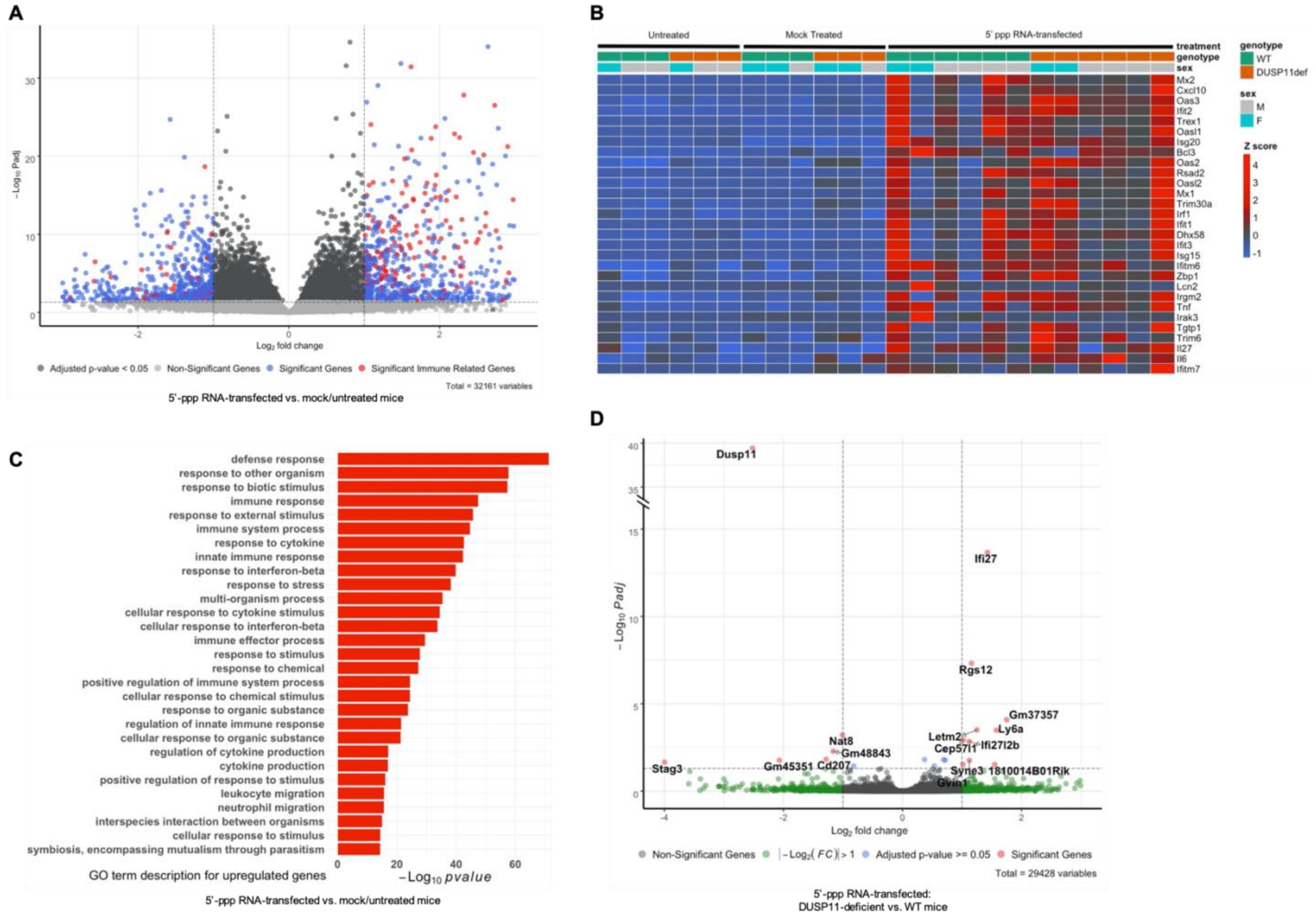

Supplemental Fig. S10. Transcriptome-wide analysis of the liver from 5'-triphosphate PAMP RNA-treated mice. (A) Volcano plot of differentially expressed genes between VSV 5'-ppp PAMP RNA-treated mice versus mock/untreated mice. Immune-related genes (gene list described in Supplemental Materials and Methods) are depicted in red. (B) Heat map of select differentially expressed genes between VSV 5'-ppp PAMP RNA-treated mice versus mock/untreated mice. Genes with adjusted p-value <=0.05, log2fc <= - 1.5 and log2fc >= 1.5 in both GO:0006955 (Immune response) and GO:0009615 (Response to virus) terms were selected. (C) Analysis of Gene Ontology (GO) Biological Process terms enriched in differentially expressed genes between VSV 5'-ppp PAMP RNA-treated mice versus mock/untreated mice. (D) Volcano plot of differentially expressed genes between VSV 5'-ppp PAMP RNA-treated DUSP11-deficient versus wild-type mice. For (A-C), liver samples from VSV 5'-ppp PAMP RNA-treated (n=12 mice: n=6 wild-type, n=6 DUSP11-deficient mice) and mock/untreated (n=12 mice: n=6 wild-type, n=6 DUSP11-deficient mice) mice were analyzed. For (D), liver samples were collected from DUSP11-deficient (n=6 mice: n=4 male, n=2 female) and wild-type (n=6 mice: n=4 male, n=2 female) mice treated with VSV 5'-ppp PAMP RNA.

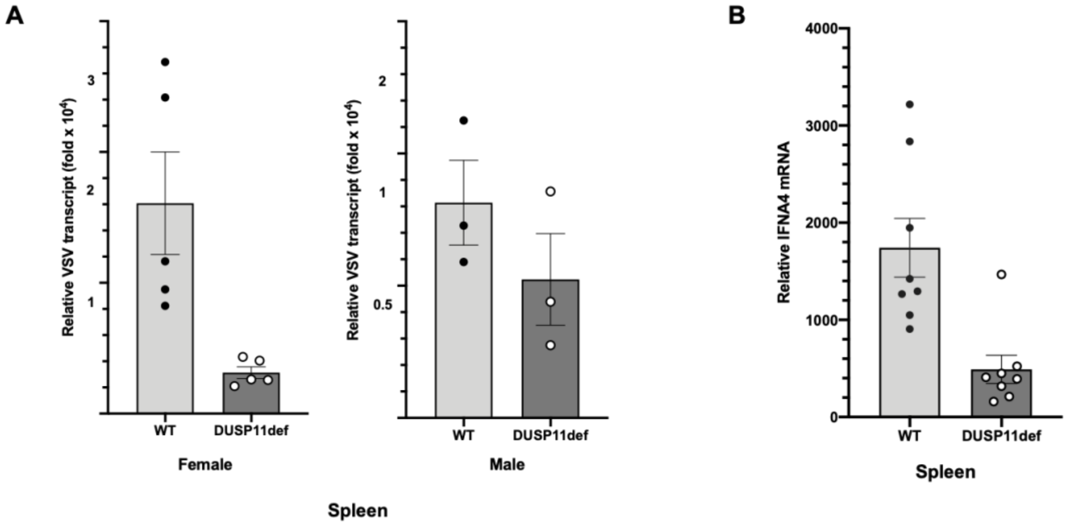

Supplemental Fig. S11. VSV replication differences in female versus male mice and interferon induction in VSV-infected mice. (A) RT-qPCR analysis of VSV transcript normalized to GAPDH mRNA in spleen RNA from VSV infected female (left) and male (right) mice. RT-qPCR results are presented relative to those of mock-treated mice. Spleen samples were collected from age/sex pair-matched DUSP11-deficient (n=3 male, n=5 female) and wild-type (n=3 male, n=5 female) mice. (B) RT-qPCR analysis of IFNA4 mRNA normalized to GAPDH mRNA in spleen RNA from VSV infected mice. RT-qPCR results are presented relative to those of mock-treated mice. Spleen samples were collected from age/sex pair-matched DUSP11-deficient (n=8 mice: n=3 male, n=5 female) and wild-type (n=8 mice: n=3 male, n=5 female) mice. Data are presented as mean ± SEM.

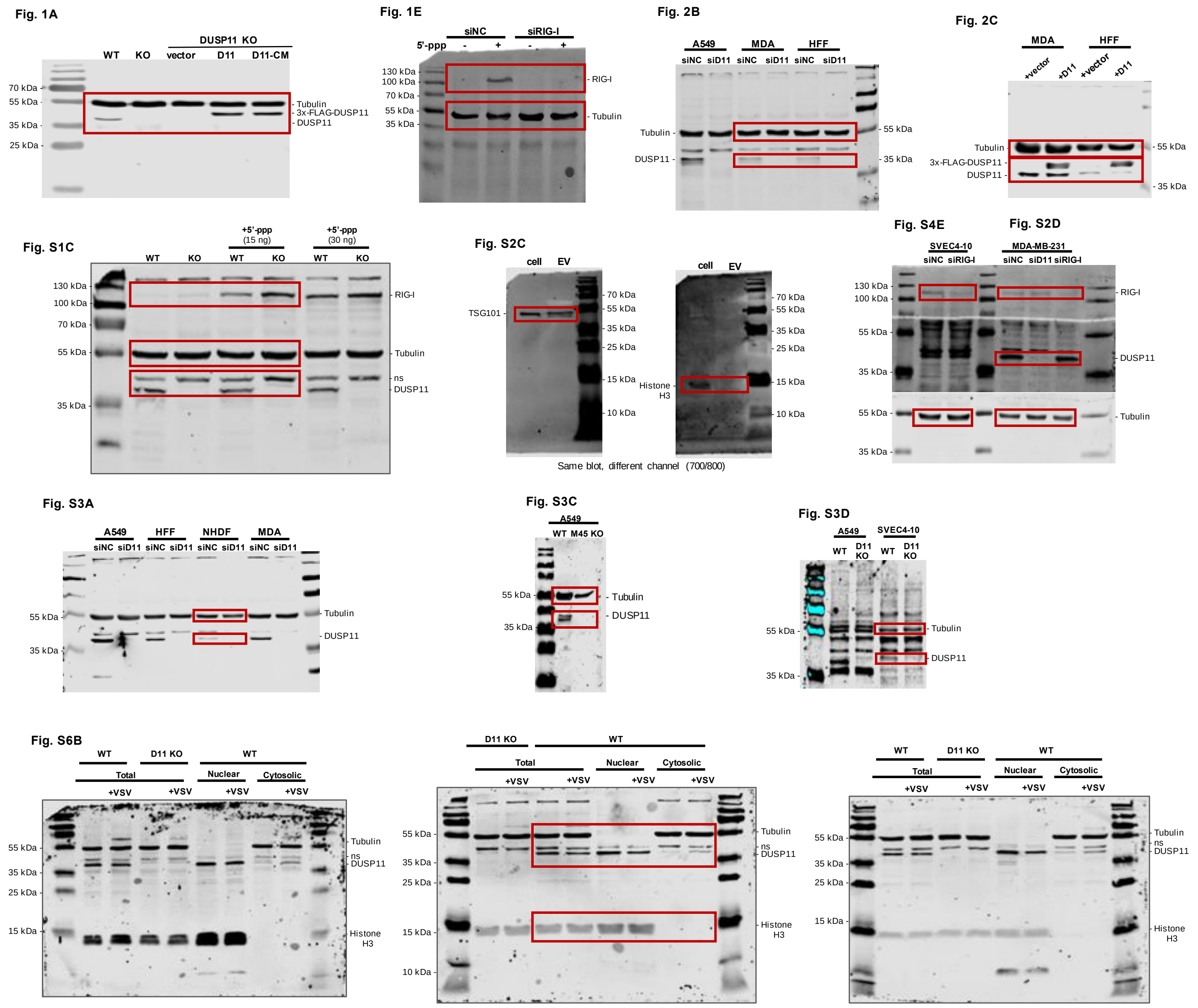

Supplemental Fig. S12. Uncropped immunoblots.

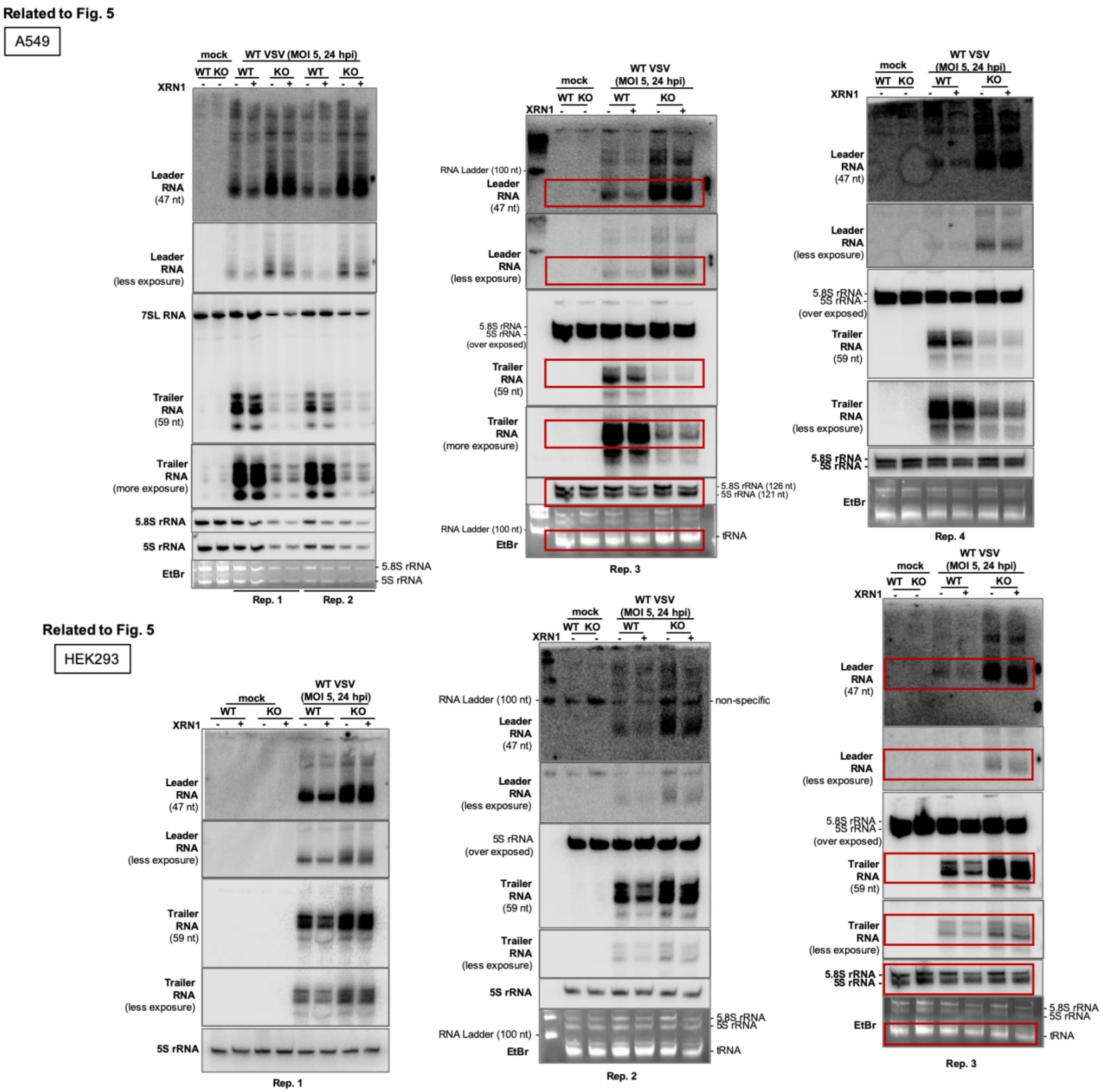

Supplemental Fig. S13. Uncropped northern blots.

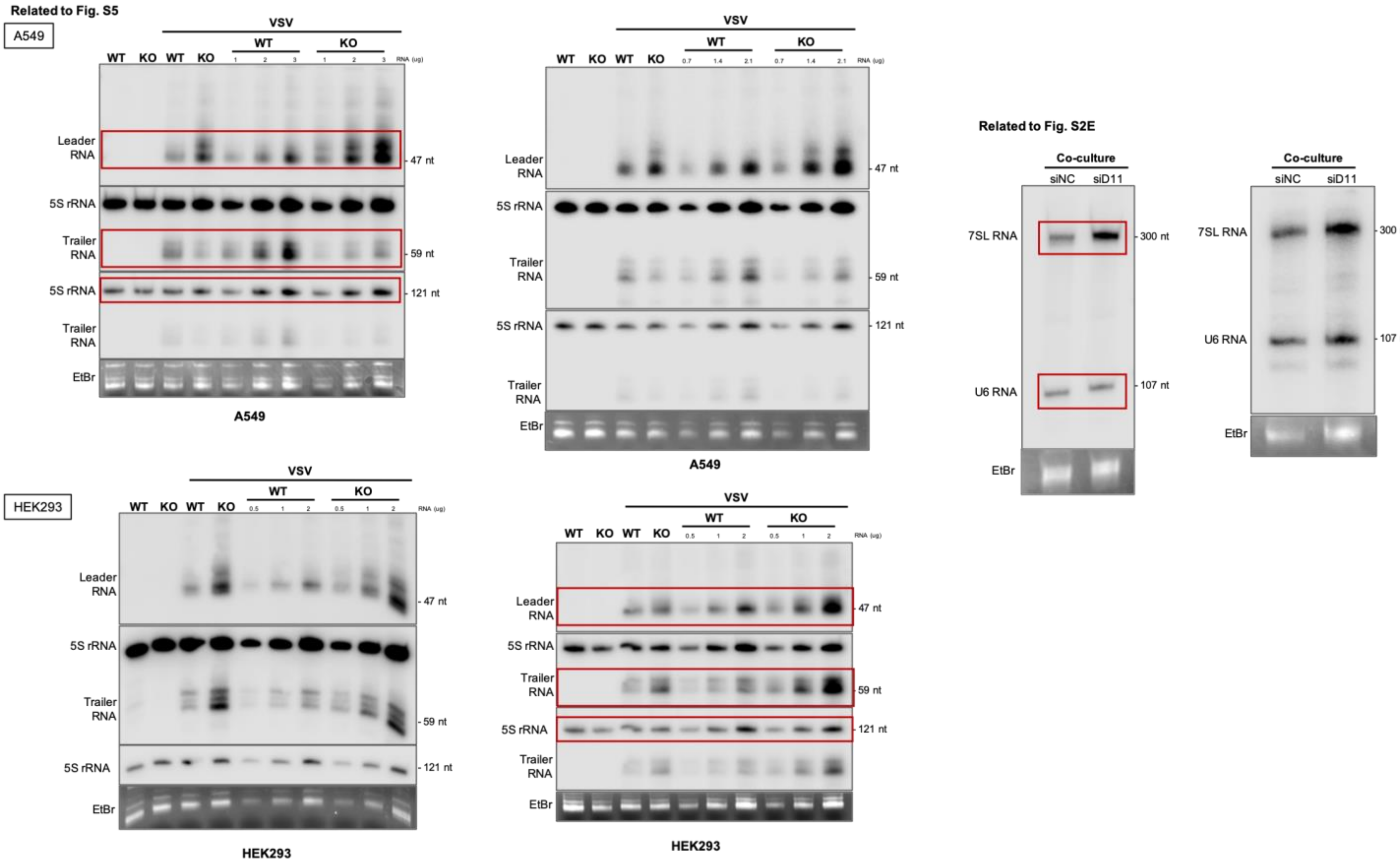

Supplemental Fig. S13. Uncropped northern blots (continued).

Supplementary Table 1. Sequence information

Northern blot analysis oligo probes	
VSV Indiana-Leader	5'- GTTTCTCCTGAGCCTTTTAATG
VSV Indiana-Trailer	5'- TCTGGTTTTGTGGTCTTCGT
7SL RNA	5'- TTTTGACCTGCTCCGTTTCCGACCT
U6 RNA	5'- CGTTCCAATTTTAGTATATGTGCTGCC
5S rRNA	5'- CCCTGCTTAGCTTCCGAGATCAGAC
5.8S rRNA	5'- TCCTGCAATTCACATTAATTCTCGCAGCTAGC

RT-qPCR SYBR Green primers (human)	
GAPDH	FWD: 5' - ACATCGCTCAGACACCATG
	FWD: 5' - ACATCGCTCAGACACCATG
IFNB1	FWD: 5' - CTCGCCAGTGAAATGATGGCT
	REV: 5'- GTCGGAGATTCGTAGCTGGAT
DDX58	FWD: 5' - TGTGGGCAATGTCATCAAAA
	REV: 5'- GAAGCACTTGCTACCTCTTGC
MX1	FWD: 5' - CGACACGAGTTCCACAAATG
	REV: 5'- AAGCCTGGCAGCTCTCTACC
ISG15	FWD: 5' - ACTCATCTTTGCCAGTACAGG
	REV: 5'- CAGCTCTGACACCGACATG
IFIT1	FWD: 5' - GGCTGCCTAATTTACAGCAACC
	REV: 5'- GGCATTTCATCGTCATCAATGG
IL-6	FWD: 5' - GGCACTGGCAGAAAACAACC
	REV: 5'- GCAAGTCTCCTCATTGAATCC
VTRNA1-3	FWD: 5' - ACTTCGCGTGTCATCAAACC
	REV: 5'- AAGAGGGCTGGAGAGCGCC
Y1	FWD: 5' - TGGTCCGAAGGTAGTGAGTTA
	REV: 5'- GTCAAGTGCAGTAGTGAGAAGG
7SL	FWD: 5' - CAAAACTCCCGTGCTGATCA
	REV: 5'- GGCTGGAGTGCAGTGGCTAT
5S	FWD: 5' - GGCCATACCACCCTGAACGC
	REV: 5'- CAGCACCCGGTATTCCCAGG
5.8S	FWD: 5' - ACTCGGCTCGTGCGTC
	REV: 5'- GCGACGCTCAGACAGG

RT-qPCR SYBR Green primers (mouse)	
GAPDH	FWD: 5' - CGTCCCGTAGACAAAATGGT
	REV: 5'- TTGATGGCAACAATCTCCAC
DUSP11	FWD: 5' - GAAAGACTATCTCCCAGTTGG
	REV: 5'- TCTGGCATAAGCTTTGCTTC
IFNA4	FWD: 5' - CTGCTACTTGGAATGCAACTC
	REV: 5'- CAGTCTTGCCAGCAAGTTGG
IFNB1	FWD: 5' - CTGGCTTCCATCATGAACAA
	REV: 5'- AGAGGGCTGTGGTGGAGAA
OAS1a	FWD: 5' - CAAGCTGAAGAGTCTCATCC
	REV: 5'- GAGCAACTCTAGGGCGTACT
IFI44	FWD: 5' - CTTGCACACAGATGATGTATG
	REV: 5'- TTCGGATGGTTTGATGTGATTG
RT-qPCR SYBR Green primers (VSV)	
VSV L	FWD: 5' - TCAGGTTGGGCCAATTACCA
	REV: 5'- GAGCCGTCTCCACAACTCAA

DUSP11 guide RNA (mouse)	5'- GCGCTTACCTTTCGGGGATGTGG

Tm1a/b mouse genotyping primers	
Ef	5' - ATTGAATTTGAGGTCTGTCAGCCAGTG
Kr	5' - CTCCTACATAGTTGGCAGTGTTTGGG
L3f2	5' - GCCTGGGACTCGTTTCATTGCTT
Lxr	5' - GAAGTTATCATTAATTGCGTTGCGCC
Tm1b_prom_F	5' - CGGTCGCTACCATTACCAGT
Floxed LR	5' - ACTGATGGCGAGCTCAGACC
Wf	5' - TTAAAACTAGATTCCACACAGG
Wr	5' - CCCATCTTGAAAACAGATAC

Table S1. Sequence information

Supplementary Table 2. RNA-seq sample metadata

Sample	Tissue	Run	SID	Treatment	Genotype	Sex	Age(weeks)	# Reads	# Aligned	% Align	Insert Size	GEO Fastq R1 file	GEO Fastq R2 file	GEO kallisto abundance file
Liver_1_01	Liver	1	1	mock	wild type	F	19	28,916,761	26,859,396	92.9%	189	Liver_1_01_R1.fastq.gz	Liver_1_01_R2.fastq.gz	Liver_1_01.transcript_abundance.tsv
Liver_1_02	Liver	1	2	mock	wild type	M	14	29,075,887	26,971,265	92.8%	188	Liver_1_02_R1.fastq.gz	Liver_1_02_R2.fastq.gz	Liver_1_02.transcript_abundance.tsv
Liver_1_03	Liver	1	3	mock	wild type	F	14	27,406,487	25,302,250	92.3%	195	Liver_1_03_R1.fastq.gz	Liver_1_03_R2.fastq.gz	Liver_1_03.transcript_abundance.tsv
Liver_1_04	Liver	1	4	mock	Dusp11 deficient	F	19	25,735,922	23,807,196	92.5%	197	Liver_1_04_R1.fastq.gz	Liver_1_04_R2.fastq.gz	Liver_1_04.transcript_abundance.tsv
Liver_1_05	Liver	1	5	mock	Dusp11 deficient	M	18	21,077,831	19,631,623	93.1%	175	Liver_1_05_R1.fastq.gz	Liver_1_05_R2.fastq.gz	Liver_1_05.transcript_abundance.tsv
Liver_1_06	Liver	1	6	mock	Dusp11 deficient	F	15	31,063,683	29,065,106	93.6%	177	Liver_1_06_R1.fastq.gz	Liver_1_06_R2.fastq.gz	Liver_1_06.transcript_abundance.tsv
Liver_2_01	Liver	2	1	transfected	wild type	M	27	25,563,791	24,082,597	94.2%	206	Liver_2_01_R1.fastq.gz	Liver_2_01_R2.fastq.gz	Liver_2_01.transcript_abundance.tsv
Liver_2_02	Liver	2	2	transfected	Dusp11 deficient	M	27	25,463,620	23,808,011	93.5%	201	Liver_2_02_R1.fastq.gz	Liver_2_02_R2.fastq.gz	Liver_2_02.transcript_abundance.tsv
Liver_2_03	Liver	2	3	transfected	wild type	M	24	25,458,053	23,906,689	93.9%	201	Liver_2_03_R1.fastq.gz	Liver_2_03_R2.fastq.gz	Liver_2_03.transcript_abundance.tsv
Liver_2_04	Liver	2	4	transfected	Dusp11 deficient	M	26	25,457,015	23,902,204	93.9%	189	Liver_2_04_R1.fastq.gz	Liver_2_04_R2.fastq.gz	Liver_2_04.transcript_abundance.tsv
Liver_2_05	Liver	2	5	transfected	wild type	F	19	25,518,438	24,042,777	94.2%	202	Liver_2_05_R1.fastq.gz	Liver_2_05_R2.fastq.gz	Liver_2_05.transcript_abundance.tsv
Liver_2_06	Liver	2	6	transfected	Dusp11 deficient	F	17	25,518,012	23,872,023	93.5%	200	Liver_2_06_R1.fastq.gz	Liver_2_06_R2.fastq.gz	Liver_2_06.transcript_abundance.tsv
Liver_2_07	Liver	2	7	transfected	wild type	M	17	24,402,232	22,954,395	94.1%	196	Liver_2_07_R1.fastq.gz	Liver_2_07_R2.fastq.gz	Liver_2_07.transcript_abundance.tsv
Liver_2_08	Liver	2	8	transfected	Dusp11 deficient	M	17	24,324,362	22,924,562	94.2%	200	Liver_2_08_R1.fastq.gz	Liver_2_08_R2.fastq.gz	Liver_2_08.transcript_abundance.tsv
Liver_2_09	Liver	2	9	transfected	wild type	F	11	25,623,266	24,013,148	93.7%	206	Liver_2_09_R1.fastq.gz	Liver_2_09_R2.fastq.gz	Liver_2_09.transcript_abundance.tsv
Liver_2_10	Liver	2	10	transfected	Dusp11 deficient	F	14	25,665,428	24,227,525	94.4%	199	Liver_2_10_R1.fastq.gz	Liver_2_10_R2.fastq.gz	Liver_2_10.transcript_abundance.tsv
Liver_2_11	Liver	2	11	transfected	wild type	M	17	24,328,501	22,988,234	94.5%	200	Liver_2_11_R1.fastq.gz	Liver_2_11_R2.fastq.gz	Liver_2_11.transcript_abundance.tsv
Liver_2_12	Liver	2	12	transfected	Dusp11 deficient	M	14	25,536,046	24,073,246	94.3%	182	Liver_2_12_R1.fastq.gz	Liver_2_12_R2.fastq.gz	Liver_2_12.transcript_abundance.tsv
Liver_3_01	Liver	3	1	untreated	Dusp11 deficient	F	23	25,370,030	23,899,186	94.2%	205	Liver_3_01_R1.fastq.gz	Liver_3_01_R2.fastq.gz	Liver_3_01.transcript_abundance.tsv
Liver_3_02	Liver	3	2	untreated	wild type	F	24	25,432,273	23,970,764	94.3%	208	Liver_3_02_R1.fastq.gz	Liver_3_02_R2.fastq.gz	Liver_3_02.transcript_abundance.tsv
Liver_3_03	Liver	3	3	untreated	Dusp11 deficient	M	21	24,291,689	22,811,317	93.9%	210	Liver_3_03_R1.fastq.gz	Liver_3_03_R2.fastq.gz	Liver_3_03.transcript_abundance.tsv
Liver_3_04	Liver	3	4	untreated	wild type	M	24	25,307,206	23,873,382	94.3%	166	Liver_3_04_R1.fastq.gz	Liver_3_04_R2.fastq.gz	Liver_3_04.transcript_abundance.tsv
Liver_3_05	Liver	3	5	untreated	Dusp11 deficient	M	24	25,376,935	23,936,080	94.3%	187	Liver_3_05_R1.fastq.gz	Liver_3_05_R2.fastq.gz	Liver_3_05.transcript_abundance.tsv
Liver_3_06	Liver	3	6	untreated	wild type	M	30	25,169,246	23,819,258	94.6%	165	Liver_3_06_R1.fastq.gz	Liver_3_06_R2.fastq.gz	Liver_3_06.transcript_abundance.tsv
Spleen_1_01	Spleen	1	1	mock	wild type	F	19	25,943,619	22,572,007	87.0%	181	Spleen_1_01_R1.fastq.gz	Spleen_1_01_R2.fastq.gz	Spleen_1_01.transcript_abundance.tsv
Spleen_1_02	Spleen	1	2	mock	wild type	M	14	27,161,183	23,594,492	86.9%	165	Spleen_1_02_R1.fastq.gz	Spleen_1_02_R2.fastq.gz	Spleen_1_02.transcript_abundance.tsv
Spleen_1_03	Spleen	1	3	mock	wild type	F	14	27,309,739	23,348,247	85.5%	185	Spleen_1_03_R1.fastq.gz	Spleen_1_03_R2.fastq.gz	Spleen_1_03.transcript_abundance.tsv
Spleen_1_04	Spleen	1	4	mock	Dusp11 deficient	F	19	24,239,223	21,261,733	87.7%	192	Spleen_1_04_R1.fastq.gz	Spleen_1_04_R2.fastq.gz	Spleen_1_04.transcript_abundance.tsv
Spleen_1_05	Spleen	1	5	mock	Dusp11 deficient	M	18	25,105,030	21,754,719	86.7%	164	Spleen_1_05_R1.fastq.gz	Spleen_1_05_R2.fastq.gz	Spleen_1_05.transcript_abundance.tsv
Spleen_1_06	Spleen	1	6	mock	Dusp11 deficient	F	15	26,537,128	23,283,561	87.7%	183	Spleen_1_06_R1.fastq.gz	Spleen_1_06_R2.fastq.gz	Spleen_1_06.transcript_abundance.tsv

Table S2. RNA-seq sample metadata

Supplemental materials and methods

Mouse genotyping

Mouse genomic DNA was extracted from tail clips collected from weaned mice using the DNeasy Blood and Tissue Kit (Qiagen). Genotyping PCR reactions were performed using Taq polymerase (New England Biolabs). Primer sets Ef/Kr and L3f2/Lxr were used to detect the Tm1a targeted allele, Ef/Kr and Tm1b_promF/FloxedLR for the Tm1b allele, and the Wf/Wr primer set for the wild-type allele (Supplemental Fig. S7) (primer sequence information in Supplemental Table S1).

Cell lines and viruses

MDA-MB-231 cells were obtained from Kevin Dalby (University of Texas at Austin, TX), primary human foreskin fibroblasts (HFF) from Vishwanath Iyer (University of Texas at Austin, TX) and SVEC4-10 cells from Jason W. Upton (Auburn University, AL). Normal human dermal fibroblasts (NHDF), A549, Vero and BHK-21 cells were obtained from ATCC. Cells were maintained in DMEM supplemented with 10% (v/v) fetal bovine serum (Corning) and 1% (v/v) penicillin-streptomycin (Corning). Use of primary HFF and NHDF cell lines were limited to less than 10 passages. Wild-type recombinant vesicular stomatitis virus (rWT-GFP VSV) and mutant VSV (rM51R-M-GFP) were provided by Douglas Lyles (Wake Forest School of Medicine, NC) and have been described previously (Ahmed et al. 2003). Wild-type VSV Indiana strain was obtained from James Pipas (University of Pittsburgh, PA). Sindbis virus Girdwood clone was provided by Mark Heise (University of North Carolina at Chapel Hill, NC) and virus stocks were prepared as previously described (Suthar et al. 2005). The HSV-1(17+) BAC strain that expresses eGFP was provided by Alistair McGregor (Texas A&M University).

Generation of CRISPR/Cas9-targeted cell lines and stable cell lines expressing recombinant DUSP11

SVEC4-10 cell lines were generated as previously described (Burke et al. 2016). In brief, Lipofectamine 2000 (Invitrogen) was used to transfect cells with hCas9 vector, human or mouse DUSP11-targeting guide RNA (gRNA) vector (mouse gRNA sequence information in Supplemental Table S1) and pLX304. 24 hours post transfection,

media was replaced with media containing 5 ug/ml Blasticidin (Invivogen) for selection. 5 days post transfection, cells were serial-diluted in 48-wells to generate individual clones. Upon propagation, clones were individually screened and validated for reduced DUSP11 protein by immunoblot analysis. When stable cell lines expressing recombinant DUSP11 were generated, lentivirus particles expressing wild-type DUSP11 or catalytic mutant DUSP11 were utilized and the transduction and selection protocols were conducted as previously described (Kincaid et al. 2013).

siRNA-mediated knockdown of DUSP11 and RIG-I

For co-culture experiments, MDA-MB-231 and HFF cells (6-well or T75 flask format, 60% confluency) were transfected with 25-35 nM of Silencer Negative Control No. 1, human DUSP11 siRNA or human RIG-I (DDX58) siRNA using Lipofectamine RNAiMAX reagent. Cells were plated for co-culture 24-48 hours post transfection. For virus infection experiments in A549 or SVEC4-10 cells (12-well format, 50% confluency), cells were transfected with 30 nM of Silencer Negative Control No. 1 siRNA, human DUSP11 siRNA or human/mouse DDX58 siRNA with Lipofectamine RNAiMAX reagent. Cells were re-plated 24-48 hours post transfection in 12-wells for a second round of siRNA transfection (50% confluency) using 10 nM of siRNA and infected with virus 24 hours post transfection. For virus infection experiments in NHDF cells (6-well format, 50% confluency), cells were treated with 40 nM negative control siRNA, 20 nM of negative control siRNA with 20 nM of DUSP11 or DDX58 siRNA, or, 20 nM DUSP11 siRNA and 20 nM DDX58 siRNA, for a total of 40 nM siRNA/well. 24 hours post transfection, 1/5 of the cells from each well were replated in a 12-well format and treated with a second round of siRNA using 40 nM of siRNA the next day. Cells were then infected with virus 24 hours post transfection. For all experiments, cell media was replaced with fresh culture medium 10-16 hours post siRNA transfection.

Extracellular vesicle isolation

Conditioned medium was centrifuged in a SX4750A rotor (Beckman Coulter) at 1,500 x g for 20 minutes to remove cells and large debris. The pellet was discarded, and the collected supernatant was centrifuged at 10,000 x g for 30 minutes in an SW-28 rotor to remove cell debris. The supernatant fraction was then centrifuged at

100,000 x g for 3 hours in an SW-28 rotor. The pooled pellet material containing EVs was collected and treated with TRIzol reagent (Invitrogen) to extract EV RNA or RIPA buffer to extract protein. To collect concentrated conditioned medium, the final supernatant fraction was centrifuged using a TLA-100.3 rotor (Beckman Coulter) at 100,000 x g for 2 hours. 1 mL of the bottom EV-concentrated fraction was used for conditioned media-transfer experiments.

5'-end characterization assay

For 7SL RNA extracted from co-culture EVs, 10 ng of EV RNA was treated with or without 0.5 ul of Terminator in a 20 ul reaction including 0.5 ul of RNase inhibitor (Thermo Fisher) and incubated for 1 hour at 30°C. Recovered RNA purified by TRIzol was subject to reverse transcription followed by qPCR. For VSV RNA 5'-end characterization, total RNA was extracted from cells infected with wild-type VSV (Indiana strain) (MOI of 5 PFU/cell, 24 hours post infection). 2-5 ug of total RNA was then treated with or without 2 ul of XRN1 in a 20 ul reaction with 1 ul of RNase inhibitor for 1 hour at 37°C. The RNA was then subject to northern blot analysis using probes specific to VSV leader and trailer RNA (Supplemental Table S1).

Northern blot analysis

Total RNA was harvested from cells using TRIzol reagent or PIG-B (Weber et al. 1998) and fractionated on a 10-12% polyacrylamide gel-electrophoresis (PAGE)-urea gel and transferred to Amersham Hybond-N+ membrane (GE Healthcare). Transferred membranes were crosslinked with a UV crosslinker (UVP) and probed in hybridization solution (Takara) with indicated DNA oligos (Supplemental Table S1) radiolabeled with [γ-^{32}P] ATP (Perkin Elmer) by T4 polynucleotide kinase (New England Biolabs). Probed membranes were exposed to a phosphor screen and visualized using Typhoon Biomolecular Imager (GE Healthcare). Membranes were stripped by incubating membrane with boiled 0.1% SDS with agitation for 15 minutes and repeated three times.

Real-time quantitative PCR

Extracted RNA was treated with DNase I (Thermo Fisher) followed by ethanol precipitation (100% ethanol, 3M sodium acetate). Complementary DNA (cDNA) was synthesized using SuperScript III reverse transcriptase (Invitrogen) using equal amounts of RNA. All RT-qPCR quantification and measurements were performed on a StepOnePlus Real-Time PCR system (Applied BioSystems). A standard 3-step PCR cycling protocol (95°C for 15 sec, 60°C for 15 sec, 68°C for 30 sec, 40 cycles) using PerfeCTa SYBR Green FastMix (Quantabio) was used according to the manufacturer's instructions.

Immunoblot analysis

Protein was extracted from cells lysed in RIPA buffer (50 mM Tris-HCl, 150 mM NaCl, 0.25% sodium-deoxycholate, 1% NP-40, 0.1% SDS with protease inhibitor (Roche)) or SDS lysis buffer (1% SDS, 2% 2-mercaptoethanol). Cell lysate was fractionated on SDS-PAGE and transferred to Amersham Protran 0.45 μm nitrocellulose membrane (GE Healthcare). DUSP11 polyclonal antibody (1:2,000 dilution, Proteintech, 10201-2-AP), RIG-I monoclonal antibody (1:2,000 dilution, Cell Signaling, 3743), α-Tubulin polyclonal antibody (1:10,000 dilution, Proteintech, 11224-1-AP), TSG101 antibody (1:5,000 dilution, Abcam, EPR7130B) and Histone H3 monoclonal antibody (1:2,000 dilution, Cell Signaling, 3638) in phosphate buffered saline with Tween 20 (PBST) was used to blot for DUSP11, RIG-I, α-Tubulin, TSG101 and Histone H3. IRDye 800CW (1:10,000 dilution, LI-COR, 926-32213) and IRDye 680LT (1:10,000 dilution, LI-COR, 926-68022) were used as secondary antibodies. Blots were washed with PBST and were imaged with an Odyssey CLx infrared imaging system (LI-COR).

Subcellular fractionation

Subcellular fraction was conducted as previously described (Castanotto et al. 2009). Briefly, cells from T25 flasks were trypsinized, resuspended in PBS and centrifuged at 3,000 rpm for 5 minutes. After aspirating the PBS, the pellet was resuspended in 150 ul of hypotonic buffer (10 mM HEPES, pH 7.9, 1.5 mM $MgCl_2$, 10 mM KCl, protease inhibitor (Roche)) and placed on ice for 5 minutes. 17 ul of the resuspension (total protein) was collected

for processing. 5.8 ul of 10% NP-40 was added to the samples, which were then gently vortexed and centrifuged at 3,000 rpm for 7 min. 60 ul of the supernatant (cytosolic fraction) was collected for processing and the remaining supernatant was aspirated. The pellet was resuspended in 100 ul of hypotonic buffer and spun down at 3,000 rpm for 5 minutes. This washing step was repeated 3 times and the remaining pellet (nuclear fraction) was further processed for immunoblot analysis.

RNA-seq data analysis

All datasets produced were 21-31 million read pairs (dataset details in Supplemental Table S2). Sequencing data quality was evaluated using the FastQC tool (ver. 0.11.9, Babraham Bioinformatics) and reports were aggregated with the MultiQC program (Ewels et al. 2016). Paired end pseudo alignment was performed against the mouse transcriptome (GENCODE ver. M24 comprehensive gene annotation set) using kallisto (ver. 0.45.0) (Bray et al. 2016) and downstream analysis was performed in R (ver. 3.4.4) (Gentleman et al. 2004). The *tximport* package (Soneson et al. 2016) was used to roll up transcript-level counts into gene-level counts provided to the DESeq2 package (ver. 1.18.1) (Love et al. 2014). Before further analysis, count data matrices were filtered to remove genes with fewer than 1 read across all included samples. In order to reduce the main source of variance determined by principal component analysis (PCA) (Supplemental Fig. S8A), separate models including model terms for sex, treatment/batch and genotype were run on data subsets. Differential expression analysis in the liver comparing wild-type and DUSP11-deficient mice was performed on the combined untreated and mock-treated datasets (Fig. 6); analyses of different treatment effects for the liver samples (mock-treated versus untreated and 5'-triphosphate RNA-treated versus un/mock-treated) were performed on corresponding data subsets (Supplemental Fig. S8B, S10A-C); analysis in the spleen comparing wild-type and DUSP11-deficient mice was performed on mock-treated mouse datasets (Supplemental Fig. S9D). Differentially expressed gene results reported are those with an adjusted p-value <= 0.05 and log2 fold change >= 1 or <= -1, unless otherwise specified. Gene ontology (GO) analyses were performed using R packages, topGO (ver. 2.34.0) and GO-MWU (Wright et al. 2015). For topGO analysis (with org.Mm.eg.db, ver 3.7.0), genes with a DESeq2 maximum adjusted p-value of 0.10 that were up-regulated (log2 fold change >= 0.5) were marked as significant for the analysis in Fig. 6C.

For analyses in Supplemental Fig. S5B and S8C, genes with a DESeq2 maximum adjusted p-value of 0.05 and log2 fold change >= 1 or <= -1 were marked as significant. The background gene universe consisted of observed genes that were assigned an adjusted p-value. The “classic” algorithm and Fisher’s exact test were used in count data mode. GO categories considered significant are those with a p-value <= 5e-5. For GO-MWU, the significance of GO categories is determined by using the Mann-Whitney U (MWU) test on the distribution of observed scores in each category. The MWU test is applied to observed scores in each GO category to obtain an individual p-value for each category; the p-values are then subjected to multiple correction testing to obtain a False Discovery Rate (FDR, or adjusted p-value). After the GO categories have been assigned FDRs, DESeq2 stat values >= 2.5 or <= -2.5 were used to count genes in the significant categories. REVIGO (Supek et al. 2011) was used to summarize representative GO subset terms using a dispensability value <= 0.75 for Fig. 6C on the top 100 terms from the topGO analysis. For Supplemental Fig. S8B and S10C, dispensability value <= 0.3 and <= 0.7 were used, respectively on the top 50 terms from the topGO analysis. To assess the overall biological relevance of differentially expressed genes (Supplemental Fig. S10A), a gene list was prepared to encompass genes involved in immune system responses. This gene list consisted of 2012 gene names from 576 GO hierarchy Biological Process terms: 573 GO terms descending from “immune system process”, and 3 “response to interferon” GO terms.

Supplemental references

Ahmed M, McKenzie MO, Puckett S, Hojnacki M, Poliquin L, Lyles DS. 2003. Ability of the Matrix Protein of Vesicular Stomatitis Virus To Suppress Beta Interferon Gene Expression Is Genetically Correlated with the Inhibition of Host RNA and Protein Synthesis. *J Virol* **77**: 4646–4657.

Bray NL, Pimentel H, Melsted P, Pachter L. 2016. Near-optimal probabilistic RNA-seq quantification. *Nat Biotechnol* **34**: 525–527.

Burke JM, Kincaid RP, Nottingham RM, Lambowitz AM, Sullivan CS. 2016. DUSP11 activity on triphosphorylated transcripts promotes Argonaute association with noncanonical viral microRNAs and regulates steady-state levels of cellular noncoding RNAs. *Genes Dev* **30**: 2076–2092.

Castanotto D, Lingeman R, Riggs AD, Rossi JJ. 2009. CRM1 mediates nuclear-cytoplasmic shuttling of mature microRNAs. *Proc Natl Acad Sci* **106**: 21655–21659.

Ewels P, Magnusson M, Lundin S, Käller M. 2016. MultiQC: summarize analysis results for multiple tools and samples in a single report. *Bioinformatics* **32**: 3047–3048.

Gentleman RC, Carey VJ, Bates DM, Bolstad B, Dettling M, Dudoit S, Ellis B, Gautier L, Ge Y, Gentry J, et al. 2004. Bioconductor: open software development for computational biology and bioinformatics. *Genome Biol* 16.

Kincaid RP, Burke JM, Cox JC, Villiers E-M de, Sullivan CS. 2013. A Human Torque Teno Virus Encodes a MicroRNA That Inhibits Interferon Signaling. *PLOS Pathog* **9**: e1003818.

Love MI, Huber W, Anders S. 2014. Moderated estimation of fold change and dispersion for RNA-seq data with DESeq2. *Genome Biol* **15**: 550.

Soneson C, Love MI, Robinson MD. 2016. Differential analyses for RNA-seq: transcript-level estimates improve gene-level inferences. *F1000Research* **4**.

Supek F, Bošnjak M, Škunca N, Šmuc T. 2011. REVIGO Summarizes and Visualizes Long Lists of Gene Ontology Terms ed. C. Gibas. *PLoS ONE* **6**: e21800.

Suthar MS, Shabman R, Madric K, Lambeth C, Heise MT. 2005. Identification of Adult Mouse Neurovirulence Determinants of the Sindbis Virus Strain AR86. *J Virol* **79**: 4219–4228.

Weber K, Bolander ME, Sarkar G. 1998. PIG-B: A homemade monophasic cocktail for the extraction of RNA. *Mol Biotechnol* **9**: 73–77.

Wright RM, Aglyamova GV, Meyer E, Matz MV. 2015. Gene expression associated with white syndromes in a reef building coral, Acropora hyacinthus. 12.
